# Cryo-EM structure of a tetrameric photosystem I from *Chroococcidiopsis* TS-821, a thermophilic, unicellular, non-heterocyst-forming cyanobacterium

**DOI:** 10.1016/j.xplc.2021.100248

**Published:** 2021-10-13

**Authors:** Dmitry A. Semchonok, Jyotirmoy Mondal, Connor J. Cooper, Katrina Schlum, Meng Li, Muhamed Amin, Carlos O.S. Sorzano, Erney Ramírez-Aportela, Panagiotis L. Kastritis, Egbert J. Boekema, Albert Guskov, Barry D. Bruce

**Affiliations:** 1 Groningen Biomolecular Sciences & Biotechnology Institute, University of Groningen, Groningen, the Netherlands; 2 Biochemistry & Cellular and Molecular Biology Department, University of Tennessee, Knoxville, TN, USA; 3 Program in Genome Science and Technology, University of Tennessee, Knoxville, TN, USA; 4 Bredesen Center for Interdisciplinary Research & Education, University of Tennessee, Knoxville, TN, USA; 5 Department of Sciences, University College Groningen, Groningen, the Netherlands; 6 Biocomputing Unit, National Center for Biotechnology (CSIC), Darwin 3, Campus Universidad Autónoma de Madrid, Cantoblanco, 28049 Madrid, Spain; 7 Universidad CEU San Pablo, Campus Urb. Montepríncipe, Boadilla del Monte, 28668 Madrid, Spain; 8 Institute of Biochemistry and Biotechnology, Martin Luther University Halle-Wittenberg, Halle/Saale, Germany; 9 Microbiology Department, University of Tennessee, Knoxville, TN, USA

**Keywords:** cryo-EM, photosystem I, non-heterocyst-forming cyanobacteria, *Chroococcidiopsis*, evolution of chloroplast, high light adaptation

## Abstract

Photosystem I (PSI) is one of two photosystems involved in oxygenic photosynthesis. PSI of cyanobacteria exists in monomeric, trimeric, and tetrameric forms, in contrast to the strictly monomeric form of PSI in plants and algae. The tetrameric organization raises questions about its structural, physiological, and evolutionary significance. Here we report the ∼3.72 Å resolution cryo-electron microscopy structure of tetrameric PSI from the thermophilic, unicellular cyanobacterium *Chroococcidiopsis* sp. TS-821. The structure resolves 44 subunits and 448 cofactor molecules. We conclude that the tetramer is arranged via two different interfaces resulting from a dimer-of-dimers organization. The localization of chlorophyll molecules permits an excitation energy pathway within and between adjacent monomers. Bioinformatics analysis reveals conserved regions in the PsaL subunit that correlate with the oligomeric state. Tetrameric PSI may function as a key evolutionary step between the trimeric and monomeric forms of PSI organization in photosynthetic organisms.

## Graphical abstract

**Figure:**
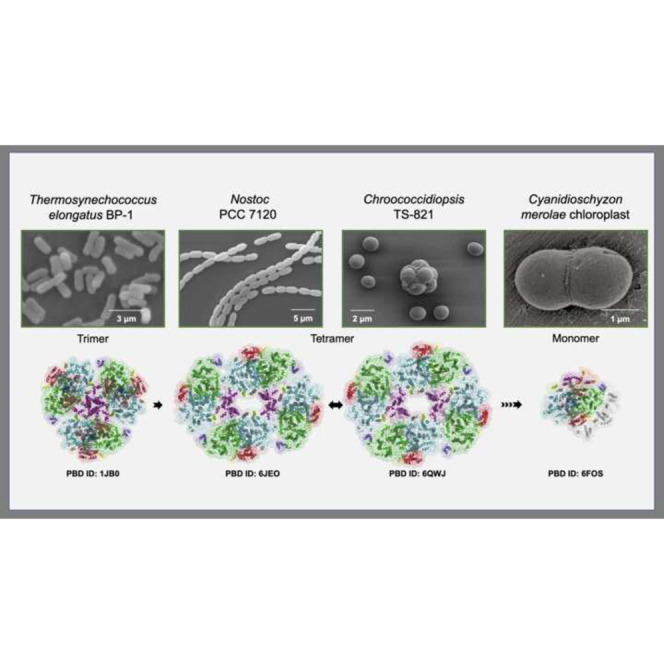

## Teaser

This article presents a ∼3.72 Å cryo-EM structure of photosystem I (PSI) from a thermophilic, non-heterocyst-forming cyanobacterium, *Chroococcidiopsis* TS-821. This second tetrameric PSI structure allows direct comparison with the three prior tetrameric and trimeric PSI structures, allowing insight into energy transfer differences. Bioinformatics analysis of PsaL offers insight into the evolution and the role of this subunit in forming different PSI oligomers.

## Introduction

Oxygenic photosynthesis is a unique energy conversion process performed by plants, algae, and cyanobacteria (Awai et al., 2014; Cardol and Krieger-Liszkay, 2017) whereby photons from sunlight are converted into chemically fixed energy by synthesizing carbohydrates, generating oxygen as a side-product of water splitting. Oxygen production and carbon dioxide fixation into organic matter performed by photosynthetic organisms determines the composition of Earth's atmosphere and provides all life forms with essential food and fuel (Nelson and Ben-Shem, 2004; Nelson and Yocum, 2006). Oxygenic photosynthesis of cyanobacteria, algae, and plants is catalyzed by four defined membrane complexes: photosystem I (PSI), photosystem II (PSII), cytochrome *b*_6_/*f* complex, and CF_1_-ATPase, which are the major components of the electron transport chain (ETC). Both PSI and PSII are large multi-subunit membrane-embedded pigment–protein complexes composed of a core complex called the reaction center, where electron transport is initiated, and a peripheral antenna system, which is essential for light harvesting and regulation of photosynthetic activity (Caffarri et al., 2014). The reaction center and peripheral antenna system work in concert to carry out the light conversion steps that ultimately lead to the production of ATP by ATP synthase and the reduction of NADP^+^ to NADPH with concomitant release of oxygen as a result of water oxidation.

The two photosystems function in series to couple the oxidation of water and enable the generation of proton motive force for ATP synthesis and reduction of NADP^+^. PSII is capable of generating powerful oxidation states that drive oxygen evolution via the water-splitting complex (Blankenship and Hartman, 1998; Umena et al., 2011). PSI is the second photosystem in the ETC. PSI includes the pair of associated chlorophylls that excite electron leaves, known as the special pair, P700, and between 96 and 112 antenna chlorophyll *a* (Chl *a*) molecules (Jordan et al., 2001; El-Mohsnawy et al., 2010) that function to increase the optical cross-section for excitation with a subsequent high-efficiency energy transfer to the special pair. Upon photoexcitation, photo-oxidized PSI transfers an electron from primary electron donor P700^+^ (a special pair of Chl *a*/*a*′ molecules) via its internal electron acceptors (A_0_, A_1_, F_X_, F_A_, and F_B_) to the terminal primarily ferredoxin (but also flavodoxin) under iron-deprived conditions (Rogers, 1987; Mondal and Bruce, 2018). In cyanobacteria, a PSI protomer comprises 12 different subunits, and in many species the total mass of a trimer is ∼1 MDa (Jordan et al., 2001; El-Mohsnawy et al., 2010; Netzer-El et al., 2019).

The structure of the cyanobacterial PSI complex has been known for nearly two decades (Jordan et al., 2001), and only recently the structures of PSI from plants and algae have been resolved (Ben-Shem et al., 2003; Mazor et al., 2017; Nagao et al., 2020; Perez-Boerema et al., 2020; Huang et al., 2021; Wang et al., 2021; Yan et al., 2021). Electron micrographs of PSI from the cyanobacterium *Synechococcus* sp. provided the first evidence for trimeric PSI over three decades ago (Boekema et al., 1987). Recently, atomic force microscopy (AFM) analysis of multiple ecotypes of *Prochlorococcus* (MacGregor-Chatwin et al., 2019) also revealed the prevalence of PSI trimers in that cyanobacterium. Further studies focused on the diverse filamentous and unicellular cyanobacteria, including the most primitive known cyanobacterium, *Gloeobacter violaceus* PCC 7421 (Boekema et al., 1987, 2001; Almog et al., 1991; Mangels et al., 2002). Eventually, the trimeric PSI structure was resolved at 2.5 Å by X-ray crystallography from the thermophilic cyanobacterium *Thermosynechococcus elongatus* BP-1 (*T*.*e*. BP-1) (PDB: 1JB0) followed by the 2.5 Å crystal structure of the mesophilic, unicellular, freshwater cyanobacterium *Synechocystis* sp. PCC 6803 (*Syn* PCC 6803) (PDB: 5OY0) (Antoshvili et al., 2019) together provided structural details of trimeric form of PSI (Jordan et al., 2001). As a result of these early seminal reports, PSI was initially believed to assemble into stable trimeric form in cyanobacteria, as opposed to the monomeric form observed in all plants and algae.

This initial belief of PSI trimer being the sole oligomeric state in cyanobacteria has recently been challenged by observation of the tetrameric form of PSI in two cyanobacteria, *Nostoc* sp. PCC 7120, also referred as *Anabaena* sp. PCC 7120 (*Nostoc*) (Kato et al., 2019; Zheng et al., 2019; Chen et al., 2020), and *Chroococcidiopsis* sp. TS-821 (TS-821) (Li et al., 2014). However, the PSI tetramer was not considered as a major oligomeric state in cyanobacteria. Although the physiological and evolutionary significance of the tetrameric state has been discussed, emphasizing the role of the lipids and PsaL subunit participation (Li et al., 2014, 2019; Kato et al., 2019; Zheng et al., 2019; Chen et al., 2020), the mechanism driving this oligomerization state and sustaining its stability in the thermophilic non-heterocyst-forming cyanobacteria remains unknown. To date, no crystal structure of the tetrameric PSI is available. Although three cryo-electron microscopy (cryo-EM) structures of the tetrameric form of PSI have been reported in the mesophilic filamentous heterocyst-forming cyanobacterium *Nostoc* (Kato et al., 2019; Zheng et al., 2019; Chen et al., 2020), our lab has observed that a tetrameric PSI organization is very prevalent, being found in most of the Heterocyst-forming cyanobacteria and their Close Relatives (HCR) after investigating 61 different cyanobacteria (Li et al., 2019). These organisms have been frequently proposed as the likely chloroplast progenitor (Dagan et al., 2013). However, other reports suggest that a nitrogen-fixing unicellular cyanobacterium similar to *Chroococcidiopsis* (or other members of the order Chroococcales) is the plastid progenitor (Falcón et al., 2011). The apparent occurrence of tetrameric PSI oligomers in all forms of cyanobacteria (mentioned above) suggests the primordial existence of tetrameric PSI form in the earliest plastid ancestor. The tetrameric form of PSI is conceivably an intermediate in the evolution of monomeric forms of PSI in algae and plants.

This work advances our understanding of the structural basis of the tetrameric form of PSI in thermophilic non-heterocyst-forming cyanobacterium by elucidating the structure of TS-821 by cryo-EM. Structure analysis visualizes the dimer-of-dimers formation, defines the correlation between the structural changes in PsaL subunit and the variations in the oligomeric state, and describes the structural relationship between the novel tetrameric PSI organization and the known trimeric one. Our study allows not only a direct comparison of the TS-821 PSI tetramer with previous trimeric PSI crystal structures from *T.e*. BP-1 and *Syn* PCC 6803, but also enables the first comparison of tetrameric PSI structures within thermophilic and mesophilic cyanobacteria. Finally, the bioinformatics analysis revealed multiple conserved regions of PsaL of TS-821 that are potentially critical for PSI oligomerization.

## Results

**Figure 1:**
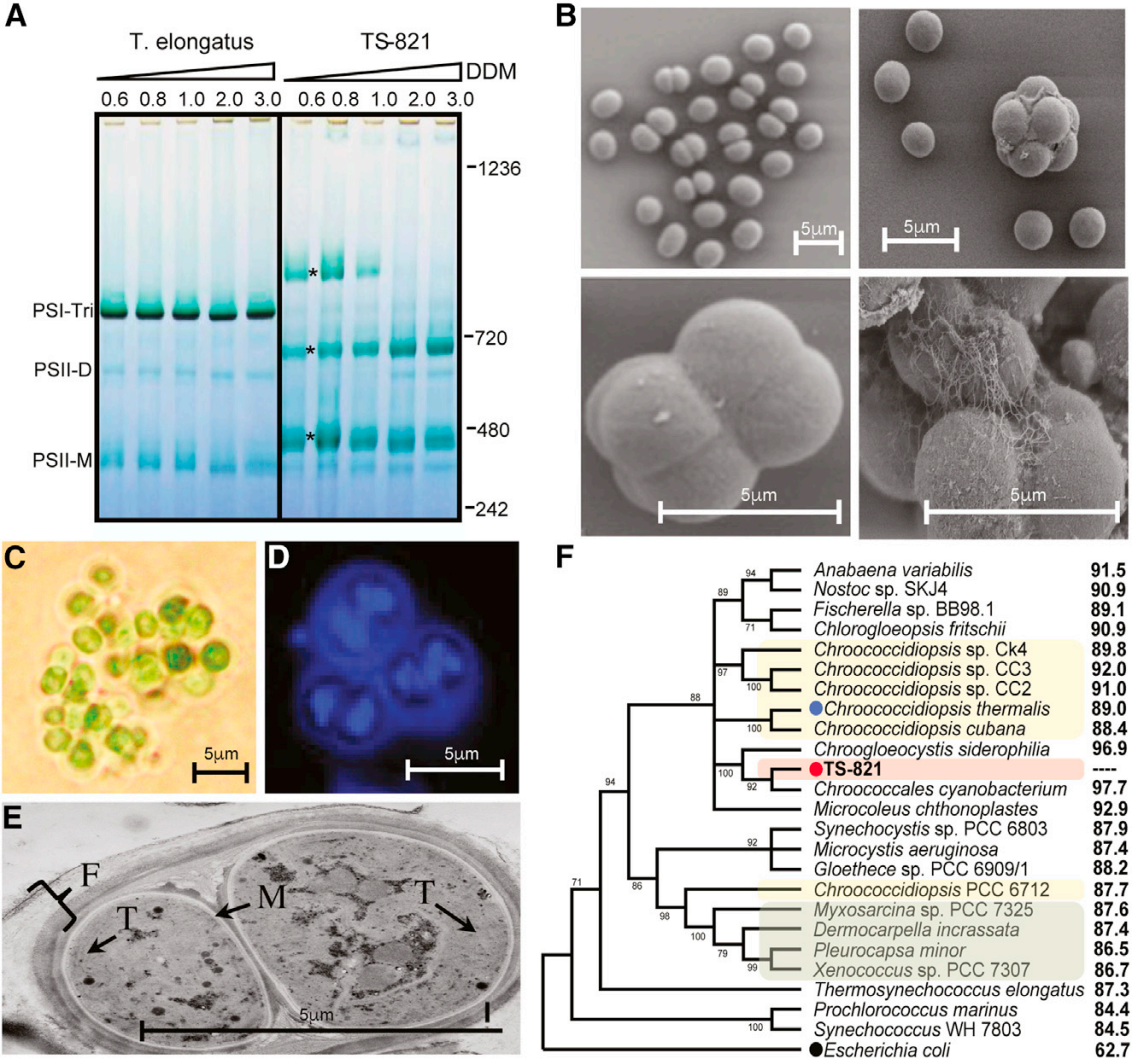
*Chroococcidiopsis* TS-821 cells and isolation of PSI. **(A)** BN-PAGE of the β-DDM solubilized thylakoids of *T*. *elongatus* (left lanes) and TS-821 (right lanes) using increasing amounts of β-DDM. The *T*. *elongatus* photosystems are identified on the left and the molecular weight standards are shown on the right. Asterisks indicate the PSI tetramer, PSI dimer, and PSI monomer (top to bottom). **(B)** Scanning electron micrographs of the TS-821 cells showing binary fission in multiple planes yielding single, dimer/octamers, and multiple or aggregate cells. **(C)** Bright-field image of TS-821 cells showing carbohydrate sheath material. **(D)** DAPI staining of the TS-821 cells showing chromosomal DNA during cell division in multiple planes, which also shows autofluorescence of extracellular sheath. **(E)** Transmission electron micrograph of a recently divided pair of cells showing the thylakoids, T; inner and outer membranes, M; and the fibrous layer (polysaccharide outer sheath), F. **(B–E)** Scale bar in each panel is in 5mm. **(F)** Phylogenetic tree based on 16S rRNA analysis: the yellow box denotes different *Chroococcidiopsis* strains, the red box is TS-821 and the green box are other members of the order Pleurocapsales. The numerical value on the right is the percent identity with TS-821. Scale bars in **(B) to (E),** 5 μm.

Initial characterization of the thylakoid membranes of TS-821 by blue native polyacrylamide gel electrophoresis (BN-PAGE) revealed a larger PSI complex (Figure 1A) (Li et al., 2019). We have extended this BN-PAGE analysis using eight other non-ionic detergents, known for their ability to maintain membrane proteins in their native conformation (data not shown), whereby we observed the same tetrameric complex. This work confirms a tetrameric form of PSI in TS-821 and argues against this form of PSI being a detergent artifact. The unicellular nature TS-821 cells by multiple imaging methods confirmed its unicellular and non-heterocyst morphology (Figure 1B–1E). These images clearly show that these cells are not filamentous yet exist either as single cells or sometimes as two-, four-, and eight-cell aggregates. The shape of these aggregates suggests that the cells undergo binary fission in multiple planes and can lead to highly symmetric octamers of cells that indicate possible coordination of cell divisions in multiple planes. The scanning electron microscopy images (Figure 1B) also revealed a thick fibrous material on the surface of the large aggregated cells. This thick fibrous material is consistent with the appearance of polysaccharides under transmission electron microscopy (TEM) (Figure 1E). TEM imaging showed a thin section of two cells revealing a thick outer sheath composed of a fibrous outer cell wall layer or F-layer, as initially observed in the *Pleurocapsales* (Waterbury and Stanier, 1978).

Observation of cells under bright-field microscopy revealed that TS-821 cells exist primarily as one or two cells or in larger aggregates (Figure 1C). Cells were also stained with 4′,6-diamidino-2-phenylindole (DAPI), a DNA-specific fluorescent dye, to observe the location of DNA within cells during division (Figure 1D). The cell undergoing binary fission exhibits fluorescence throughout the entire interior portion of the cell, suggesting that DNA is distributed throughout the cell (Figure 1D). However, distinct regions of fluorescence were seen within adjacent cells dividing via multiple fissions. These distinct globular regions of DNA are most likely small daughter cells that result from multiple fissions. Together, this imaging supports the original morphological classification of TS-821 as a member of the order Pleurocapsales (Waterbury and Stanier, 1978).

To analyze the phylogeny of strain TS-821, we generated a phylogenetic tree as described in materials and methods. It is also important to note that genera representing all major orders within the phylum Cyanophyta are included within this phylogenetic analysis. Figure 1F shows the resulting unrooted consensus tree with branches containing bootstrap values where values below 50% were excluded. Bootstrap values are interpreted as the confidence level in the branch's arrangement for which they are listed. Phylogenetic analysis of TS-821 revealed that TS-821 does not group close to the other *Chroococcidiopsis* sp., but rather with other unclassified cyanobacteria, with high confidence. However, it is distant from other members of the Pleurocapsales, and the phylogenetic 16S tree indicates that TS-821 is much closer to heterocyst-forming cyanobacteria such as *Nostoc* and *Fischerella* as previously reported (Li et al., 2014). At the same time, the 16S sequence strongly suggests that it is distinct from the other more well-characterized members of the *Chroococcidiopsis* genus and may require reclassification in the future.

### Cryo-EM and model building

Although our earlier cryo-EM low-resolution structure suggested that TS-821 tetramer was a dimer-of-dimers, the 11.5 Å resolution prevented us from investigating the structural basis of this unique symmetry (Semchonok et al., 2016). To obtain a better insight into the organization of tetrameric PSI from TS-821, we conducted single-particle cryo-EM analysis, including single-particle two-dimensional (2D) classification and three-dimensional (3D) model reconstruction (Supplemental Figure 1A–1C). From 4845 micrographs we extracted 325 648 particles that we used for further 2D classification. This 2D classification yielded 16 different 2D classes (Supplemental Figure 1B). We then subjected the good set of particles to 3D classification, reducing the number of particles to 122 411. This set of particles was then subjected to several 3D refinements with Xmipp highres, ending up with 63 130 good particles. Further map refinement proceeded with RELION 3.0 3D auto-refinement, using the previous 3D projection map from Xmipp highres as a reference. The final resolution of ∼3.72 Å (Fourier shell correlation [FSC] 0.143) of the resulting cryo-EM 3D map was obtained (Supplemental Figure 2A).

The local resolution of the final 3D map varies from 3.2 Å to 5.5 Å (Supplemental Figure 2B) with high resolution within the transmembrane core of each monomer composed of multiple PsaA and PsaB helices, suggesting increased protein stability and less conformational flexibility. In addition, the interfacial subunits PsaL, PsaI, and PsaM at one of the dimeric interfaces contain the best resolution distribution within the map, possibly reflecting higher stability.

To reconstruct a tetramer of PSI, we manually placed the single protomer of cyanobacterial PSI (PDB: 1JB0) in the cryo-EM map using Chimera (Pettersen et al., 2004). This rough placement was followed by rigid-body refinement of each subunit in Phenix (Liebschner et al., 2019). We mapped density for all known PSI subunits except for PsaX, which had a very fragmented and weak density. This could indicate that this subunit may have been partially lost during the sample preparation. Thus, PsaX was not included in further modeling. The sequence was manually adjusted during the modeling in Coot (Emsley and Cowtan, 2004). The positions of Chl *a* molecules from the 1JB0 structure were used as reference points to model Chl *a* in the tetramer. The map resolution was not sufficient to model the positions of carotenoids. The manually rebuilt model underwent several rounds of real-space refinement in Phenix (Liebschner et al., 2019), including non-crystallographic symmetry (NCS) restraints, secondary structure restraints, and simulated annealing protocol. The positions of side chains were modeled according to the density, except for the ambiguous situations, where the most common rotamers were utilized.

### Overall structure and placement of subunits

**Figure 2:**
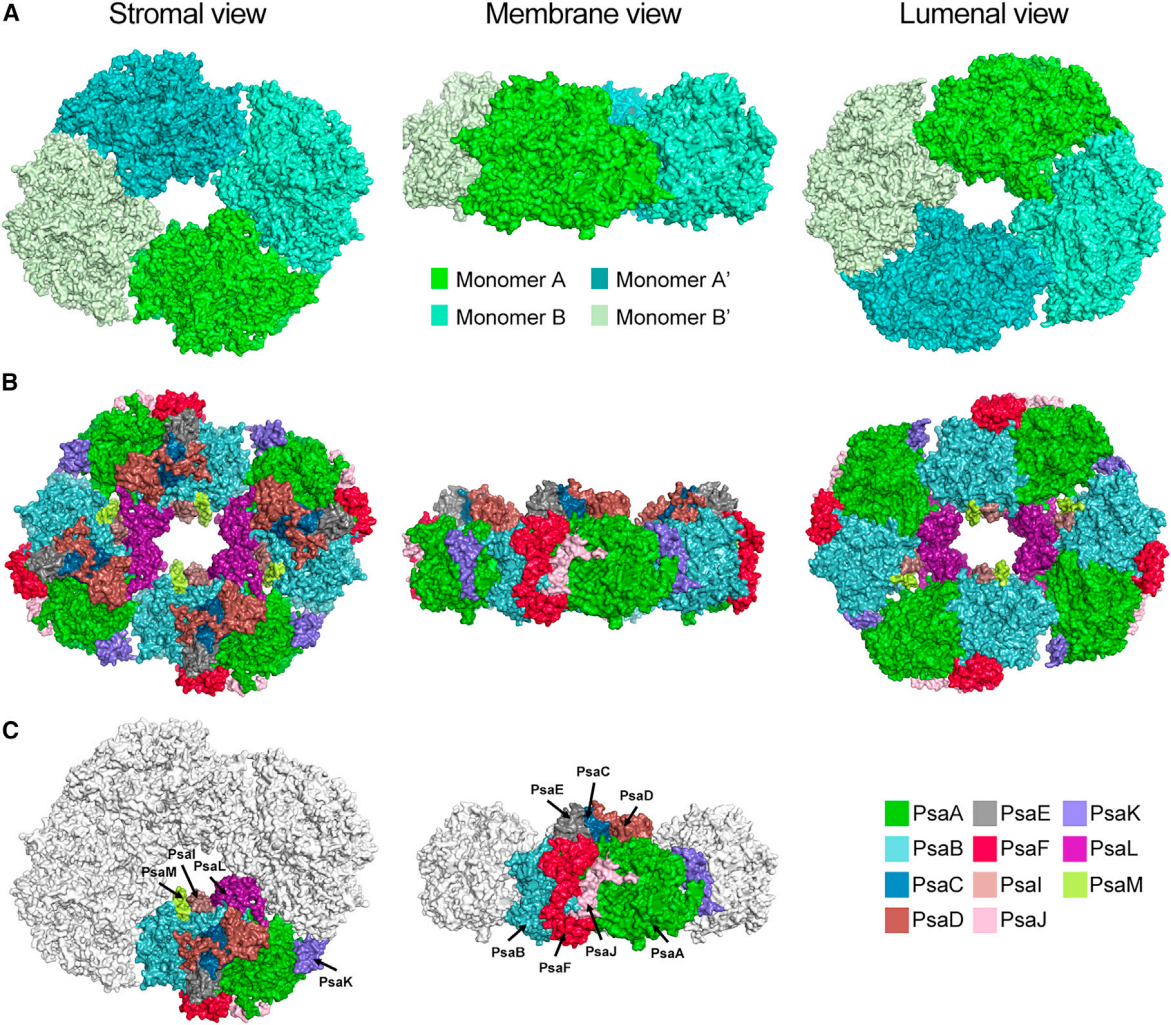
Tetrameric PSI structure of TS-821. **(A)** Surface view of PSI monomers (A, B, A′, and B′) that make up the tetramer are shown in stromal (left), membrane (center), and lumenal (right) views. Each monomer is identical but colored differently for the purpose of visualization. **(B)** Stromal (left), membrane (center), and lumenal (right) view of tetrameric PSI colored by chain. **(C)** Labeled subunits of one of the monomers are shown in membrane (top) and stromal (bottom) view.

As we have previously shown (Semchonok et al., 2016), the tetramer is organized as a dimer-of-dimers with two different interfaces: A–B and B–A′ (Figure 2A). Therefore, the structure is pseudotetrameric and has C2 and not C4 symmetry. The obtained cryo-EM structure of the tetrameric PSI of TS-821 resolves 44 subunits: four each of PsaA, PsaB, PsaC, PsaD, PsaE, PsaF, PsaI, PsaJ, PsaK, PsaL, and PsaM (Figure 2B and 2C). The inner cavity is surrounded by four PsaL, two PsaM, and two PsaI subunits. The relative positions of the subunits are identical in the individual monomers. However, the tetramer has a dual symmetry where the positioning of the monomers A and B is identical to that of monomers A′ and B′, supporting the hypothesis that tetrameric PSI is a dimer-of-dimers. The PsaL subunits are closely associated with each other between monomers A–B and A′–B′ (Figure 2B). Interestingly, PsaL subunits in adjacent monomers B–B′ and A–A′ are not in contact with each other. PsaM and PsaI subunits of monomers A and A′ are oriented toward the inside cavity of the tetramer. In monomers B and B′ these two subunits are buried in the interface region between monomers A–B and A′–B′.

### Differential stability of dimeric interfaces

The two different dimeric interfaces, A–B and B–A′, are shown in the lumenal and stromal view of Supplemental Figure 3A and 3B with the interfacial amino acid residues shown in space-filling. An enlarged view of these interfaces from the external membrane view is shown in Supplemental Figure 3B. The residues contributing most to the stabilization of each interface are shown as spheres and also denoted in Supplemental Figure 3C. To qualitatively assess the stability of these interfaces, we performed refinement of each interface with HADDOCK (Dominguez et al., 2003), including parameterization of Chl *a* molecules. The results reveal a differential contribution of combined non-covalent forces governing each interface (Supplemental Figure 3C and 3D). Although the overall calculated energetics are similar in the HADDOCK score, electrostatic contributions are substantial in the A–B′ interface as compared with the A–B interface. Thus, charge–charge interactions are of more considerable significance for A–B′. The decreased contribution of electrostatic energies is compensated by extensive van der Waals interactions in the A–B interface (Supplemental Figure 3C). The A–B interface contains more proximal Chl (11) than the B–A′ interface (6). However, the B–A′ interface includes more transmembrane domains (14) than the A–B interface (7). This compositional difference is reflected in the increased buried surface area of A–B (2418 Å^2^) whereas B–A′ only has 1561 Å^2^ (Supplemental Figure 3D). In addition, we compared interfaces with missing Chl *a* molecules to assess the impact of the Chl *a* molecules. Overall, Chl *a* molecules contribute to the solvation energies of both interfaces and the regulation of electrostatic energies. As expected, they also contribute to the larger surface area for the A–B and A′–B′ interfaces, indicating that they are integral for their formation and stability.

Similar results were obtained by using Monte Carlo calculations (Metropolis and Ulam, 1949) to allow the sampling of amino acids' protonation states. To understand the stability of the interfaces between the different monomers, we extracted the amino acids that have their Cα within 12 Å from the amino acids of corresponding monomers. Hence, Monte Carlo sampling was used to obtain Boltzmann distribution for the protonation patterns. We then evaluated the electrostatic and van der Waals interactions between 78 and 119 amino acids for interfaces A–B′ and A–B, respectively, based on Boltzmann occupancies (Supplemental Figure 3D). The sum of the Coulombic interactions between the amino acids in interface A–B′ is −0.4 kcal/mol, while the sum of van der Waals interactions is −14 kcal/mol. For interface A–B, the contribution from Coulombic and van der Waals interactions is 0.1 kcal/mol and −16 kcal/mol, respectively. These results indicate that the structure of the dimer-of-dimers is mainly stabilized by the dispersion interactions (London forces).

### Cofactor placements

Based on the densities and our molecular modeling, we are able to place all of the known light-harvesting Chls and electron transport components. There are three [4Fe-4S] clusters (F_A_, F_B,_ and F_X_) in each monomer. F_A_ is located in PsaA subunit, and both F_X_ and F_B_ are located in the PsaC subunit. In the resolution of our model, we could not unambiguously identify any carotenoids or lipids and therefore not deposited in the PDB dataset. However, based on unresolved densities and comparative analysis of the carotenoids in the *T*.*e*. BP-1 PSI crystal structure (PDB: 1JB0), we have been able to putatively place both phylloquinones and carotenoids in our model. Each monomer has two phylloquinones. At this resolution the specific identity of the carotenoids could not be matched to the previously chemically identified carotenoids (Li et al., 2019) and are thus modeled as β-carotene (BCR). We compared the BCR placement with the *Nostoc* PSI tetramer (PDB: 6JEO) and identified differences in the two species (Supplemental Figure 4). Even though most BCRs are in locations identical to those in *Nostoc*, there are additional BCRs noted in *Nostoc* in almost all monomers, whereas in the case of TS-821 there is one additional BCR in monomer B and two BCRs in monomer B′ (Supplemental Figure 4A). Overall, there are 18 BCR molecules in monomers A and A′ while there are 20 in monomer B and B′. There are a total of 358 Chl *a* molecules in the tetramer, 91 in monomer A, 89 in monomer B, 90 in monomer A′, and 88 in monomer B′ (Supplemental Figure 5). The special pair of Chl *a* is located at the center of the PsaA–PsaB interface, toward the lumenal side. Closely placed Chl *a* molecules with Mg^2+^–Mg^2+^ distance <10 Å are found primarily within individual monomers but are not found in any of the four interfaces between monomers (Supplemental Figure 6A). A pair of Chl *a* molecules has one Mg^2+^–Mg^2+^ distance of less than 15 Å at both the A–B and A′–B′ interfaces (Supplemental Figure 6B). There are no Chl *a* molecules with Mg^2+^–Mg^2+^ distances <15 Å across the B–A′ and B′–A interfaces (Supplemental Figure 6B). There are many Chls within <20 Å in all the interface regions (Supplemental Figure 6C). Supplemental Figure 6D shows all of these contacts superimposed. These closely placed Chl pairs are delineated by their monomer, chain ID, and residue ID in Supplemental Table 2. Key Chl residues at the monomer interfaces are highlighted in Supplemental Table 2 and are shown in Supplemental Figure 6E. In monomers B and B′ there are two Chl *a*s oriented parallel to one another near the monomer interface (Supplemental Figure 6E). Five lipids (1,2-dipalmitoyl-phosphatidylglycerol, LHG) were putatively placed in our model and compared with the lipids identified in the *Nostoc* tetramer (Chen et al., 2020) (Supplemental Figure 7). In *Nostoc*, there are three LHGs in each monomer placed in identical locations. Out of the five LHGs of TS-821 PSI aligned with those of *Nostoc* PSI, two each were found in monomers A and A′ while only one was found in monomer B′.

### Central cavity

**Figure 3:**
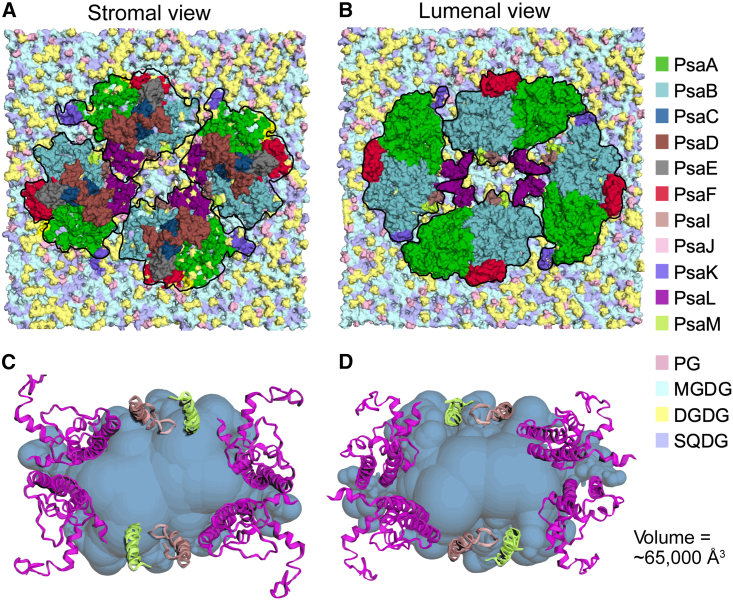
Potential lipid capacity and central cavity size. **(A and B) (A)** Stromal and **(B)** lumenal surface views of tetrameric PSI embedded in a model cyanobacterial membrane generated with CHARMM-GUI and embedded with VMD. The membrane contains 47% MGDG, 23% DGDG, 21% SQDG, and 9% PG. **(C and D) (C)** Stromal and **(D)** lumenal views of the central cavity (blue) calculated with CASTp. Only chains that form the cavity are shown (PsaL on all four monomers, PsaI and PsaM on monomers B and B′).

The central cavity of the tetramer contains unresolved densities which may correspond to lipids, detergent molecules, or pigment/carotenoid molecules. A model cyanobacterial membrane containing 47% monogalactosyldiacylglycerol (MGDG), 23% digalactosyldiacylglycerol (DGDG), 21% sulfoquinovosyl diacylglycerol (SQDG), and 9% phosphatidylglycerol (PG) was generated with CHARMM-GUI. The tetrameric PSI was embedded in the membrane using VMD (Figure 3A and 3B). CASTp (Tian et al., 2018b) was used to calculate the volume of the central cavity using PsaI, PsaL, and PsaM subunits. The central cavity is about 70 × 50 Å and has an approximate volume of 65 000 Å^3^ (Figure 3C and 3D). Although we do not observe densities in this central cavity, the volume would suggest it could accommodate a bilayer with about 25–30 lipids per leaflet. Prior work has shown that the TS-821 tetramer is enriched in novel carotenoids (Li et al., 2014; Kato et al., 2019; Zheng et al., 2019; Chen et al., 2020) that are lost upon detergent destruction into dimers, which may suggest that this central cavity contains carotenoids that may be lost during isolation.

### Differences in structure with trimeric PSI

**Figure 4:**
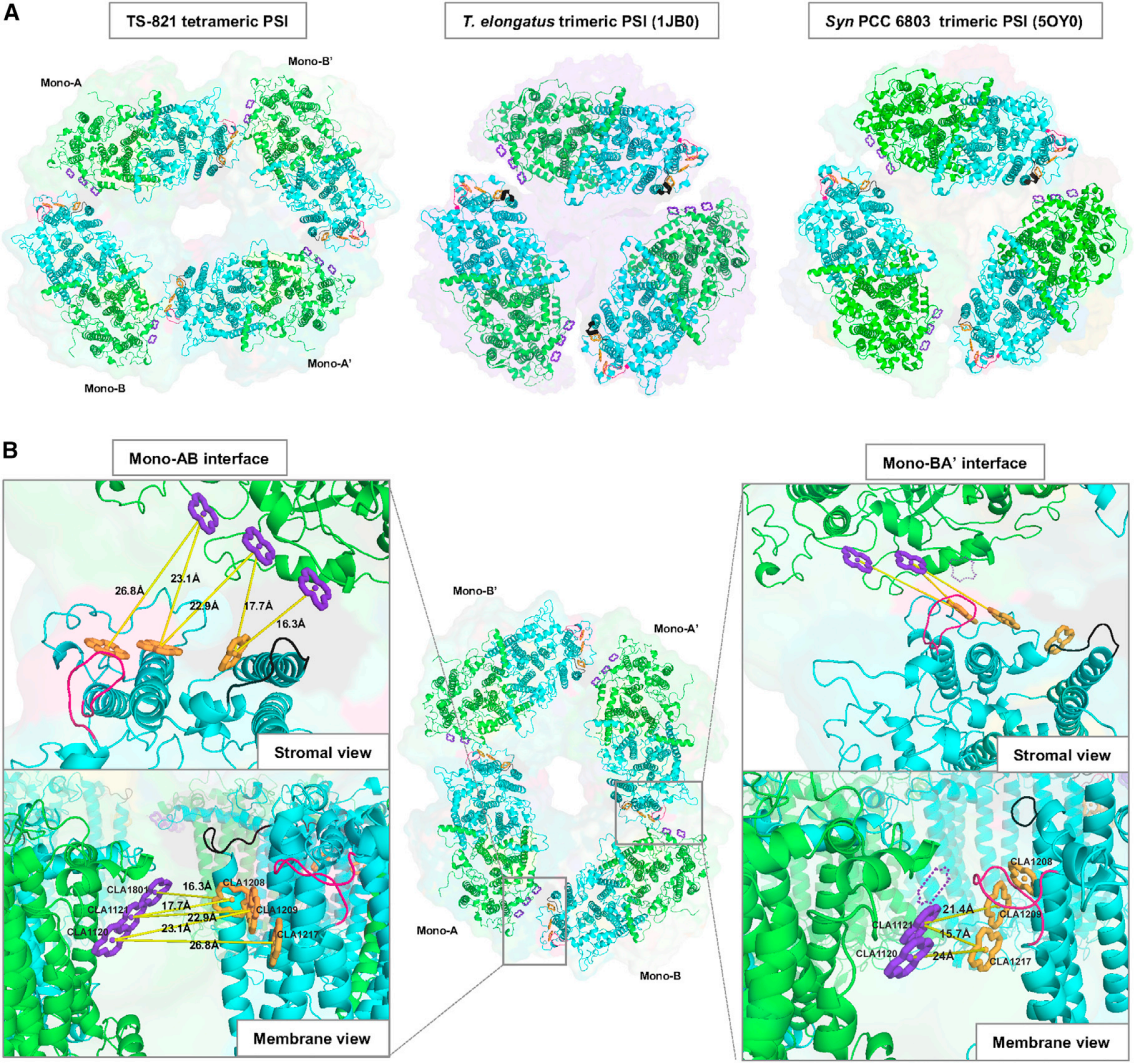
Interface Chl *a*s between PSI monomers of TS-821 tetrameric PSI versus *T.e*. BP-1 trimeric PSI and PCC 6803 trimeric PSI. **(A)** Tetrameric PSI of TS-821 (left), trimeric PSI of *T.e*. BP-1 (PDB: 1JB0) (center), and trimeric PSI of *Syn* PCC 6083 (PDB: 5OY0) are shown in surface view; only chain PsaA (green) and PsaB (blue) are shown as cartoon. In all three structures, the pyrrole rings of Chl *a* associated with PsaA at the interface of the PSI monomers are highlighted in purple and those associated with PsaB are highlighted in orange. **(B)** A closer look of the interfaces: in B′–A and A–B, the black and red loops are part of PsaB with sequences QPKFRPS and MYRTNFGIGHS, respectively. The distances are shown by yellow lines. The missing Chl *a* at the B–A′ interface is shown by a purple dashed pyrrole ring at a position relatively similar to that in the mono-A–B interface.

To describe the differences in Chl placements in the monomeric interfaces of the tetrameric PSI from the earlier elucidated trimeric structures, we directly compared TS-821 PSI with *T*.*e*. BP-1 (PDB: 1JB0) and *Syn* PCC 6803 PSI (PDB: 5OY0) crystal structures (Figure 4 and Supplemental Figure 8). These Chls may function in the energy transfer between adjacent PSI monomers. Interestingly, only the A–B and A′–B′ interfaces are conserved in contacts and packing to the three identical interfaces found in the trimeric PSI (Figure 4A). The enlarged view in Figure 4B reveals the three parallel Chl *a* molecules observed at these interfaces of tetrameric PSI from TS-821 that are also observed in the trimeric PSI complexes. However, the other dimer interfaces in the tetramer (A–B′ and A′–B) form a distinct interface that is not observed in the interface regions of either trimeric PSI. These Chl *a* molecules are located much closer together at the A–B and A′–B′ interfaces in the tetramer as compared with the A–B′ and A′–B interfaces. The larger surface area of the A–B and A′–B′ interfaces along with the conservation of the Chl *a* arrangement with trimeric structures suggests that these two interfaces likely form the dimers and that the A–B′ and A′–B interfaces are responsible for joining the two dimers together.

### Role of PsaL in interface stability

**Figure 5:**
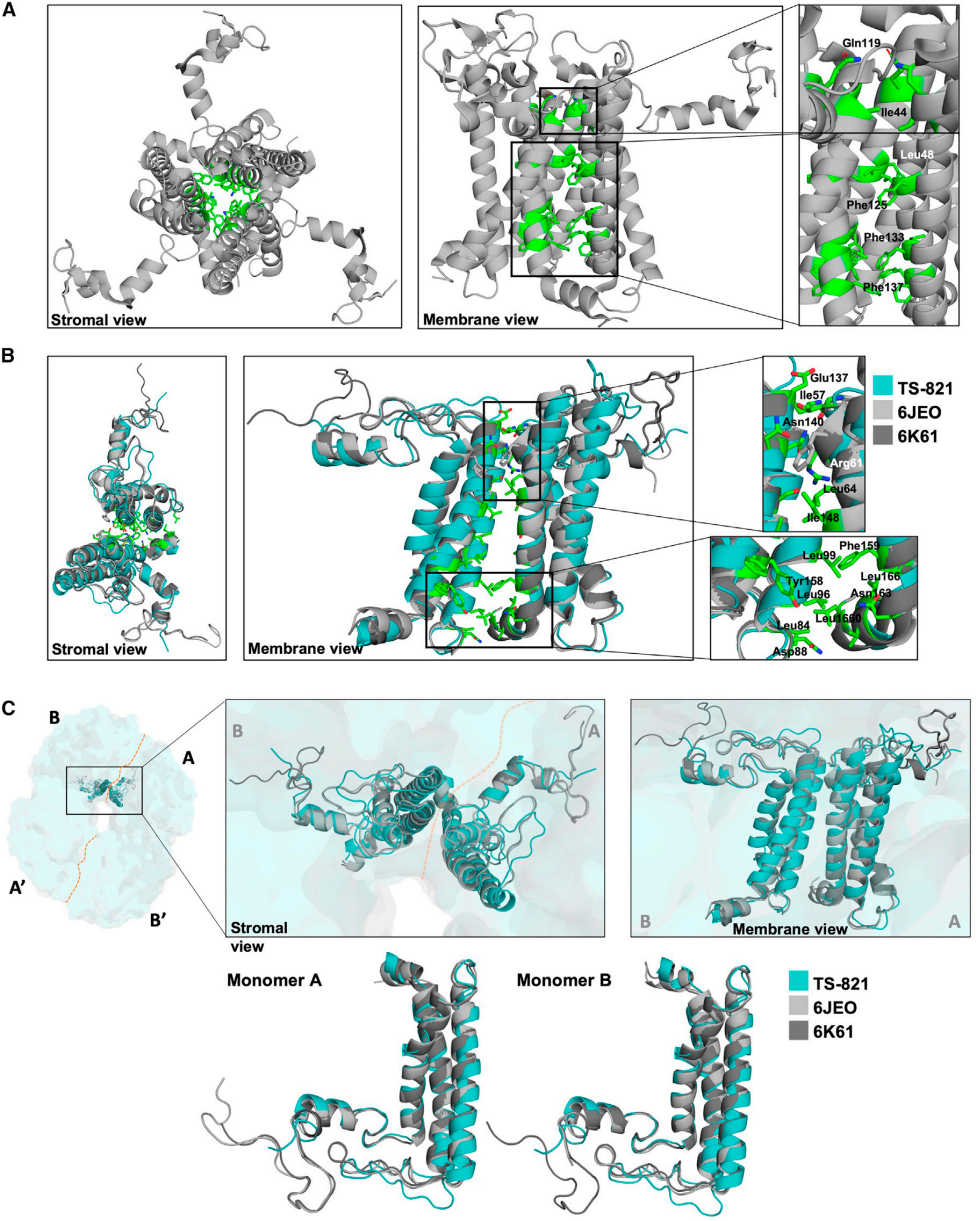
Interaction interface of PsaL subunits in trimeric (*T.e*. BP1) and tetrameric (TS-821, PDB: 6JEO and 6K61) PSI. **(A)** Polar and non-polar residues that participate in the interaction interface at the interface of the three PsaLs of their respective monomers in *T.e*. BP-1. **(B)** Residues participating in the interaction interface between the two PsaLs of the A–B or A′–B′ interface, root-mean-square deviation (RMSD) 0.708 (6JEO and 6K61 aligned) and RMSD 5.732 (TS-821 and 6JEO aligned). **(C)** PsaL from all three tetramers’ (TS-821, 6JEO, and 6K61) A–B interface (stromal and membrane view). The aligned PsaL from both monomers A and B are also shown at the bottom.

Early work has shown that PsaL is key to the assembly and stability of the trimeric structure of PSI (Chitnis and Chitnis, 1993). Since the A–B interface in the tetrameric PSI resembles the trimeric PSI interface at the core, we investigated the interaction interface of PsaLs in PSIs of both *T*.*e*. BP-1 and TS-821 and other tetramers (PDB: 6JEO and 6K61) (Figure 5). The three PsaL subunits form a central helical bundle in *T.e*. BP-1 PSI trimer (Figure 5A). This central core contains a PsaL subunit from each PSI monomer and has virtually no cavity. In the trimer, the enlarged view highlights the residues that stabilize this PsaL bundle. These residues span the entire transmembrane region with mostly non-polar interactions. However, the dimeric PsaL bundle in all three tetramers superimposed (PDB: 6QWJ, 6JEO, and 6K61) shown in Figure 5B and 5C reveals interactions only at the lumenal and stromal regions. At the PsaL interface (A–B and A′–B′) of the tetramer, only the lumenal face has a few hydrophobic residues present (Figure 5B). Interestingly, there is a large number of polar side chains in the stromal and lumenal faces at the PsaL interface of the tetramer.

### Evolutionary differences in PsaL

**Figure 6:**
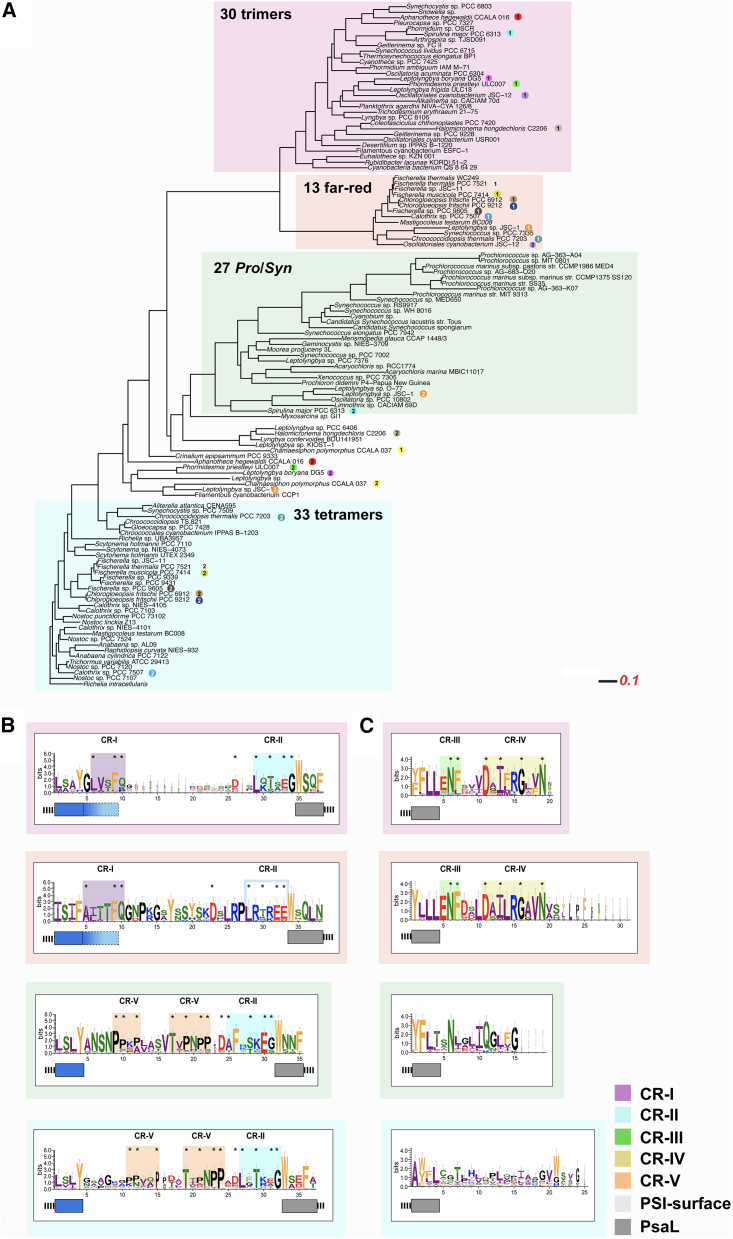
Phylogenetic analysis of PsaL and motif analysis. **(A)** Representation of a maximum-likelihood tree built using FastTree 2 on multiple alignments of the 108 PsaL proteins using MUSCLE. The color-shaded regions delineate separate groups that are known to include different forms of PSI including trimeric forms of PSI, far-red light forms of PSI, tetrameric forms of PSI, and the fourth group of marine cyanobacteria including members of *Prochlorococcus* and *Synechococcus*. Some species contain multiple copies of PsaL in their genome, which are denoted by colored circles (by species) and numbers (by the number of PsaL copies). **(B)** Logo plot of the loop region between the predicted TMD #2 and #3. The ending and beginning of the TMD are shown by colored boxes below the Logo sequence. The bit score scale was set to 6 bits to allow the error bars to be visible. The conserved motifs were shaded and named CR-I to CR-V. Within each CR the most conserved amino acids are further indicated by an asterisk above the single-letter code. **(C)** Similar to (B), the Logo plots of the PsaL C terminus of the four different groups are shown, also indicating the conserved regions (CR-III and CR-IV).

Our prior work has highlighted that all or nearly all of the HCR contain a tetrameric form of PSI (Kato et al., 2019; Li et al., 2019; Zheng et al., 2019). Since previous work has shown that PsaL is driving the trimerization (Chitnis and Chitnis, 1993), is not clear what determines the oligomeric state of the PSI tetrameric complex. However, prior work has identified changes in two regions of the PsaL primary sequence that seem to correlate with trimeric and tetrameric forms (Li et al., 2019). To investigate this in greater detail, we compared 113 PsaL orthologs from a broad range of cyanobacteria including those shown to be trimeric, far-red-light-inducible Chl *f*-containing, marine (*Prochlorococcus*/*Synechococcus*), and putative tetrameric cyanobacteria. This phylogenetic approach is both broader and based on a different evolutionary trait from either the initial tree based on only 16S rRNA (Figure 1A) or the previous tree utilizing 29 universally conserved genes (Li et al., 2019). Moreover, this phylogenetic tree is focused on changes associated with PsaL (Figure 6A).

Our analysis of a non-redundant set of PsaL sequences yielded four putative monophyletic clusters (Figure 6A). Each cluster does contain multiple species whose PSI oligomeric state/type is experimentally known (trimeric, tetrameric, or far-red), giving us further confidence in this phylogenetic approach to identify PSI oligomerization states. In some cases, organisms contained multiple PsaL proteins that fit into two or three groups. Classification of each group was based on existing structural data from representative members: using *Leptolyngbya* sp. strain JSC-1 (Gan et al., 2014) as an anchor for the far-red cluster, *T.e*. BP-1 as trimer cluster (Jordan et al., 2001), and *Chroococcidiopsis* sp. TS-821 as tetramer anchor (Li et al., 2014, 2019), the *Prochlorococcus/Synechococcus* group does not yet have a high-resolution structure; however, a recent AFM study clearly indicated a trimeric form of PSI (MacGregor-Chatwin et al., 2019). Upon this agnostic phylogenetic classification, we identified some sequence/structure variations found within the PsaL gene when each PSI cluster identified in Figure 6A was analyzed by a LogoPlot of the region between transmembrane domain (TMD) #2 and #3 (Figure 6B) as well as the C-terminal region (Figure 6C). Both of these regions were previously identified as variable regions of PsaL in cyanobacteria (Li et al., 2014).

The sequence LogoPlots of the trimer and far-red sequences are most alike based on conserved regions (CRs) across CR-I and CR-II in the linker region (Figure 6B). Similarly, the far-red and trimers show signs of common ancestry based on the C-terminal regions, especially CR-III and CR-IV. Interestingly, the tetramers were most similar to the *Prochlorococcus*/*Synechococcus* group based on their linker region with the conserved TV/T/APNPP motif found in the CR-V region (Figure 6C). Unlike the trimers and far-red groups, there were no CRs between the tetramers and the *Prochlorococcus*/*Synechococcus* group across the C-terminal regions. Three *Prochlorococcus* genomes of SS120 (low light), MIT9313 (low light), and MED4 (high light) clustered with the *Prochlorococcus*/*Synechococcus* group even though they are known to form trimeric PSI structures (MacGregor-Chatwin et al., 2019).

**Figure 7:**
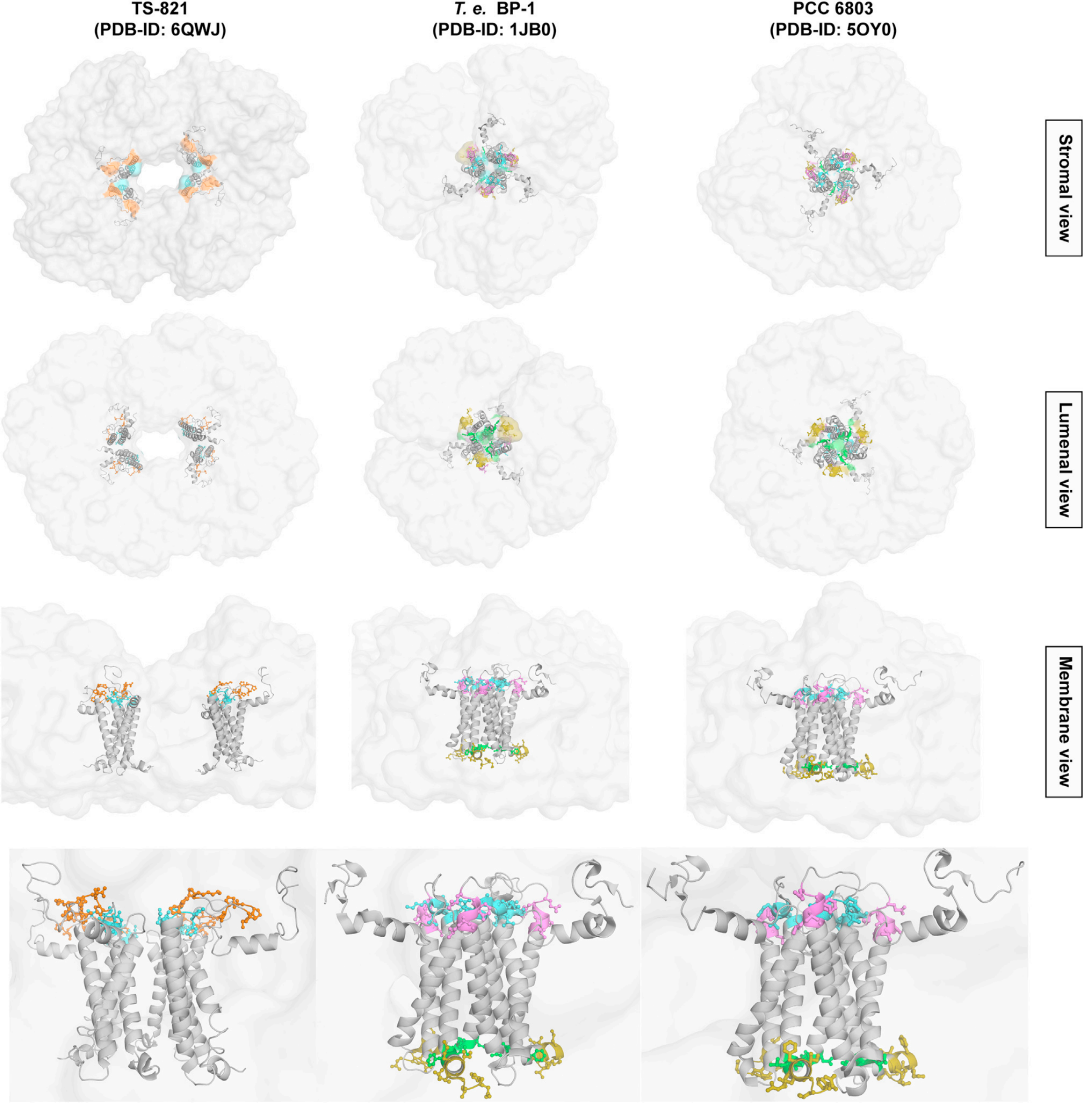
Comparison of the PsaL conserved regions in TS-821, *T.e*. BP-1, and *Syn* PCC 6803 PSIs. The CRs (as highlighted in Figure 6B and 6C) are shown from different perspectives: stromal (top row), lumenal (second row), membrane (third row), and enlarged membrane (bottom row) view.

The LogoPlot identified CRs (CR-I to CR-V) spatially placed in the known structures of the tetrameric TS-821 PSI and the trimeric PSI structures from *T.e*. BP-1 and *Syn* PCC 6803 (Figure 7). It is clear that these CRs form different associations based on the oligomeric state. Inspection of these models reveals apparent differences in where these CRs reside in the different PSI oligomers. For example, in the tetramer, CR-II and CR-V are located in the stromal face of both A–B and A′–B′ interfaces. In trimeric PSI, CR-I and CR-II are located in the stromal face while CR-III and CR-IV are located in the lumenal face. CR-II is found in both the tetrameric (in the dimeric interfaces A–B and A′–B′) and the trimeric PSI (central core), which suggests a crucial role of this region in oligomerization of PSI dimer in the case of tetrameric and trimeric PSI. On the other hand, CR-III and CR-IV are only found in trimeric and far-red PSIs, which suggests a sole role in trimeric PSI. Interestingly, CR-III and CR-IV are not observed in the marine cyanobacteria (*Prochlorococcus*/*Synechococcus*), raising questions as to the oligomeric state of PSI in these organisms. This is supported by the confirmation and placement of CR-II and CR-V, both the tetramers and the marine PsaLs having two copies of CR-V.

## Discussion

In this study, the structure of tetrameric PSI from TS-821, a thermophilic non-heterocyst-forming cyanobacterium, was solved by cryo-EM with a resolution of 3.72 Å and has an organization of a dimer-of-dimers. The tetramer exhibits two types of interfaces: A–B (between monomer A and B) and B–A′ (between monomer B and A′). The A–B interface of the tetramer closely resembles that of the trimeric PSI of other cyanobacteria (*T*.*e*. BP-1 and *Syn* PCC 6803) whereas the B–A′ interface is novel and resembles the two reports of a tetrameric PSI in *Nostoc* sp. PCC 7120, a mesophilic filamentous heterocyst-forming cyanobacterium (Kato et al., 2019; Zheng et al., 2019) (see alignment in Supplemental Figure 9). This suggests that the tetrameric form of PSI is widespread, occurring in both heterocyst-forming and non-heterocyst-forming cyanobacteria. The Chls in both tetramers show distinct arrangements at the two interfaces as compared with the trimeric PSI. The Chl placements in the A–B interface are more similar to that observed at the trimeric interfaces but are clearly different from the Chl positions at the B–A′ interface. The three parallel Chls common to the A–B interface and trimer suggest that this dimeric interface most resembles the trimeric interface. Our extended phylogenetic analysis of PsaL confirms our prior observation that changes in the loop between TMD #2 and #3 and the C terminus may have an evolutionary and structural role in how subunit PsaL alters the oligomeric state of PSI. Structural analysis suggests that the PsaL core in trimeric PSI compared with that of tetramer have multiple bulky residues, mostly aromatic residues all along the TMD. In the tetramer, only the lumenal face of PsaL at the A–B interface has multiple bulky (mostly non-polar and aromatic) residues, while the stromal face has polar residues. This is similar to that of the tetrameric PSI of heterocyst-forming cyanobacteria, for which it was suggested that specific amino acids with large side chains might prevent the formation of the trimer due to the steric hindrance provided by these bulky groups (Kato et al., 2019).

Our work adds a new tetrameric PSI cryo-EM structure for a cyanobacterium outside of the three reports in *Nostoc* sp. PCC 7120 (Kato et al., 2019; Zheng et al., 2019; Chen et al., 2020). This work, coupled with our prior BN-PAGE and bioinformatics characterization (Li et al., 2014; Semchonok et al., 2016) suggest that most, if not all members of the HCR group of cyanobacteria have a tetrameric form of PSI. However, the evolutionary role of this change in PSI structure is still elusive. Previous work has shown that in three different cyanobacteria, exposure to high light can induce the formation of tetrameric PSI and was also shown to induce the accumulation of more novel carotenoids in the thylakoid membranes (Li et al., 2019). This might suggest that one role of the tetramer is to allow accumulation of photoprotective carotenoids with PSI when exposed to high-light environments. How these carotenoids are associated with PSI is unknown, but their release upon dissociation into two dimers suggests that one possibility is an association within the ∼65 000 Å^3^ central cavity. This higher carotinoid amount can also be a part of a thermoadaptation mechanism that helps to support the photosynthetic reactions under high temperatures (Mandelli et al., 2012, 2017).

The subunit PsaL was found to be essential for the formation of the trimeric form of PSI (Chitnis and Chitnis, 1993). Our bioinformatics work has identified small CRs in PsaL that correlate with this tetrameric symmetry by promoting PsaL dimerization versus a trimerization in the PSI timers. According to the study by Netzer-El et al. (2019), the C-terminal region of PsaL creates a short helix that associates with the PsaL subunits of the neighboring PSI monomers in the trimer, assisting in trimer stabilization; however, addition of a terminal histidine disrupts this association resulting in a largely monomeric form of PSI along with dislocation and structural differences in subunits PsaM and PsaI, which are located in the trimerization region. In our study, we highlight the placement of PsaL in the formation of two dimeric interfaces and how the protein–protein interactions at these interfaces are quite different from those observed to stabilize the trimeric form. Phylogenetic analysis, along with motif analysis, has revealed several loosely conserved regions (CR-I to CR-V) within the PsaL subunit that contribute to oligomerization of PSI tetramers. Our analysis suggests that cyanobacteria with strictly trimeric PSI along with far-red-type PSI trimers have common ancestry with specific CRs. On the other hand, the PsaL from marine cyanobacteria (*Prochlorococcus*/*Synechococcus*) and those that have tetrameric PSI lack CR-III and CR-IV but has a new conserved domain, CR-V. The presence of semi-conserved CR-II in all four groups suggests its role in all PSI structures, yet this region is the least conserved overall. These general observations may be complicated since some organisms encode multiple copies of the PsaL genes, yet in most cases each PsaL is placed phylogenetically within one of the four groups.

Our bioinformatics analysis of the PsaL protein of most *Prochlorococcus* strains (including MIT9313 and MED) did not cluster with trimeric group in the PsaL tree, yet was placed much closer to the tetrameric PSI-forming cyanobacteria. Surprisingly, a LogoPlot of the loop region between TMD #2 and three of the marine cyanobacteria identified CR-V that is enriched in Pro residues. There are actually two CR-V motifs that are also conserved with species known to have the tetrameric form of PSI. Although the structure of PSI from these marine cyanobacteria have not been studied by crystallography or cryo-EM, there was a recent AFM study of two *Prochlorococcus* ecotypes including high-light (MED4) and low-light (MIT9313) ecotypes (MacGregor-Chatwin et al., 2019). Using AFM imaging of intact thylakoids, this group observed that non-trimeric forms of PSI (dimeric/monomeric) were significantly increased (∼5-fold) relative to PSI trimers when the cells were grown in high-light conditions. This suggests that either the formation of trimers or the stability of existing PSI trimers is reduced upon high-light exposure. Together, this suggests that although the thylakoids of the *Prochlorococcus*/*Synechococcus* group may contain PSI trimers, upon exposure to high light there is a major shift toward dimeric/monomeric forms of PSI.

Our biochemical observation of tetrameric PSI in the HCR may reflect increased stability of the tetramer, possibly due to the high local concentration of PSI and/or the lipid composition of the thylakoid region or subdomain where PSI is localized. TS-821 is a thermophilic cyanobacterium that will have predominantly saturated fatty acids, possibly because of limited fatty acid desaturase genes (FAD) as observed in *T. elongatus* (Chi et al., 2008). However, the marine cyanobacteria (*Prochlorococcus*/*Synechococcus*) are mesophiles and have been shown to have highly unsaturated fatty acids due to their multiple FAD genes (Chi et al., 2008; Breton et al., 2020) and possibly because of the recently identified activity of cyanophage-encoded lipid desaturases (Roitman et al., 2018). Future work will be needed to find out whether the marine cyanobacteria contain a stable dimeric or tetrameric form of PSI when grown in high light.

Early analysis of 56 different cyanobacteria suggested that chloroplasts arose as a single monophyletic event from an organism that is most closely related to N_2_-fixing unicellular cyanobacteria (Chroococcales) and possibly their sister group of closely related heterocyst-forming cyanobacteria (Nostocales) ∼2.1 billion years ago (Falcón et al., 2011). It has been shown that members of the genus *Chroococcidiopsis* are the closest living relatives to the filamentous heterocyst-differentiating cyanobacteria (Fewer et al., 2002). We and others have shown that the genus *Chroococcidiopsis* includes species with unique survival abilities under nitrogen-limiting conditions and can grow fairly well in salt water (Fewer et al., 2002). These authors speculated that *Chroococcidiopsis* was capable of surviving, following a sudden washout, into an increasingly saline environment, thereby providing a route for the evolution of open, ocean-dwelling cyanobacterial strains (Herrmann and Gehringer, 2019). Recently, Sanchez-Baracaldo et al. (2017) proposed that the closest relative of the chloroplast was an ancient freshwater cyanobacterium, *Gloeomargarita*. Their data suggest that the ancestor of the chloroplast and *Gloeomargarita* diverged about 2.1 billion years ago, which is relatively early in the evolutionary timeline of the cyanobacterial lineage (de Vries and Archibald, 2017; Sanchez-Baracaldo et al., 2017) and prior to when planktonic marine cyanobacteria diverged from freshwater ancestors (Sanchez-Baracaldo, 2015). It will be interesting to discover whether *Gloeomargarita* has a tetrameric form of PSI, which may suggest that it could give rise to both marine cyanobacteria and chloroplasts. This early placement of a tetrameric PSI in cyanobacterial and possibly chloroplast evolution is supported by the observation that a tetrameric PSI was also found in *Cyanophora paradoxa*, a member of the Archaeplastida and the most primitive group of photosynthetic eukaryotes known (Watanabe et al., 2011). Collectively, this work suggests that tetrameric PSI is a widely occurring form of PSI that may be an adaptation to high-light conditions during cyanobacteria expansion and maybe the key intermediate in the evolution of PSI structure in plants and algae.

## Materials and methods

### Source of TS-821 cyanobacterium

*Chroococcidiopsis* TS-821 was originally isolated over 25 years ago from the Sankampaeng and Mac Fang hot springs near Chiang Mai in the northern part of Thailand (Hayashi et al., 1994, 1997). All strains had vegetative cells surrounded by fibrous (F) layers. Early analysis by light and electron microscopy revealed that cell division occurred by binary fission, but neither motility nor mobile baeocytes were observed. Based on work by Woodbury and Stainier, TS-821 (Waterbury and Stanier, 1978) was assumed to belong to the genus *Chroococcidiopsis*. Further early work indicated that TS-821 was able to overgrow to dense cultures and was proposed to be a source for early biomass conversion (Hayashi et al., 1995, 1997).

### Growth of cyanobacteria

*Chroococcidiopsis* sp. TS-821 (TS-821) was cultured in a 2-l glass culture bottle with aeration at 45°C, with continuous white fluorescent light of ∼40 μmol/m^2^/s at the bottle surface. Cells were harvested at the late log phase, and the wet cell mass harvested from a 2-l bottle culture is usually 3–4 g.

### Cell lysis and isolation of cyanobacterial thylakoids

Thylakoid membrane isolation was carried out similarly to earlier studies (Watanabe et al., 2011; Li et al., 2014). Homogenized suspensions of cells in ice-cold lysis buffer (50 mM 2-(*N-morpholino)ethanesulfonic acid [*MES]–NaOH [pH 6.5], 5 mM CaCl_2_, 10 mM MgCl_2_, 0.5 M sorbitol) were ruptured by passing through the French press three times at 15 000 psi. After removing the intact cells by centrifugation at 10 000 *g* for 5 min, the thylakoid membrane was pelleted after 30 min at 193 000 *g* (Type 50.2 Ti, Beckman) centrifugation. The pelleted membranes were washed in wash buffer (50 mM MES–NaOH [pH 6.5], 5 mM CaCl_2_, 10 mM MgCl_2_) and pelleted again before final resuspension in storage buffer (50 mM MES–NaOH [pH 6.5], 5 mM CaCl_2_, 10 mM MgCl_2_, 12.5% [v/v] glycerol) and homogenized before storing at −20°C or immediate solubilization.

### Isolation of PSI tetramers

The TS-821 thylakoid membrane containing 1 mg/ml Chl *a* were solubilized in 1% β-DDM (Glycon Biochemicals, Luckenwalde, Germany). The solubilized membrane solution was loaded on a 10%–30% sucrose gradient containing 0.01% β-DDM in the wash buffer. Two-step ultracentrifugation was used to purify the PSI tetramer as described previously (Li et al., 2014) except for the following modifications. The first centrifugation was done using at 30 000 rpm (SW 32 Ti, Beckman) for 20 h. The concentrated and dialyzed PSI tetramer from the first spin was loaded on a 10%–30% sucrose gradient again and centrifuged at 30 000 rpm for 24 h. PSI tetramers from the second gradient after spinning were dialyzed and concentrated for analysis.

### DAPI imaging of TS-821 cells

DAPI staining was used in order to observe DNA within intact cells. This staining allowed determination of the state of fission—binary or multiple—a particular cell was undergoing as well as the location of the dense nucleoid regions. Two microliters of DAPI stain was added to 1 ml of liquid cell culture, covered with aluminum foil, and incubated at room temperature with shaking for 1 h. The sample was observed using a Nikon Eclipse 80i fluorescent microscope under the DAPI filter.

### Scanning electron microscopy

The samples were fixed in 3% glutaraldehyde buffered with 0.1 M sodium cacodylate. Following a 1-h incubation period, the samples were washed in cacodylate buffer three times, allowing 10 min per wash, and post-fixed in cacodylate buffered with 2% osmium tetroxide for 1 h. Samples were then washed three times with deionized water. During the final wash, small aliquots of the sample were allowed to settle onto a 2 × 3-mm silicon chip, which had been previously washed with polylysine. The sample was then dehydrated in a graded acetone series and critical point dried in CO_2_ using a Ladd Research Critical Point Dryer. Dried samples were coated with gold with an SPI Sputter coater before examination in a Zeiss 1525 scanning electron microscope.

### Transmission electron microscopy

The fixation protocol is the same as described above until the dehydration step. Samples were then washed in water three times before dehydration in a graded ethanol series, and finally dehydrated with propylene. Samples were then embedded in Spurr epoxy for 48 h prior to the final embedding and subsequent curing of the resin at 68°C for 24 h. For ultramicrotomy, samples were sectioned with a Reichert OMU3. Thin sections of approximately 70–90 nm were stained with methanolic uranyl acetate and lead citrate before examination in a Hitachi H800 transmission electron microscope operating at 75 keV. Images were recorded on Kodak 4489 electron microscopic film.

### DNA isolation and cloning of 16S RNA

DNA isolation was performed using a modification of the Saha et al. (2005) protocol. This method was modified to use mechanical cell lysis using a FastPrep-24 Tissue and Cell Homogenizer and Orange Capped Lysis Matrix A tubes, which are specific for DNA isolation. A large cell pellet harvested from dense liquid cell culture was resuspended in 1 ml of 1× TE buffer (10 mM Tris–HCl [pH 7.5], 1 mM EDTA) and homogenized at 4 m/s for 20 s. Lysate appeared blue due to the release of phycobilin proteins into solution. This tube was centrifuged for 1 min at 10 000 *g*, and the supernatant was immediately transferred to another1.5-ml microfuge tube. All additional steps were done as previously described (Saha et al., 2005). Due to a significant amount of RNA contamination, 3 μl of RNase (10 mg/ml) was added to each tube and allowed to incubate at room temperature for 10 min. Amplification of the 16S rRNA was accomplished using these primers: forward (5′-AGA GTT TGA TCC TGG CTC AG-3′) and reverse (5′-AAG GAG GTG ATC CAR CCG CA-3′). The 50-μl reaction mixture consisted of 10 μl of 5× GoTaq Reaction Buffer, 1 μl of dNTP mixture (10 mM each), 1 μl of each primer (10 pM/μl), 0.25 μl of GoTaq Polymerase, 50 ng of template DNA, and an appropriate amount of nuclease-free water to achieve 50 μl total reaction. The PCR conditions were as follows: 95°C for 3 min, 30 cycles of 95°C for 1 min, 60°C for 1 min, 72°C for 1.5 min, and finally 72°C for 10 min subsequently followed by a 4°C hold. The resultant PCR product was cleaned up using QIAquick PCR clean-up system and immediately ligated into the TOPO 2.1 (Invitrogen) vector as directed and transformed into TOP10 chemically competent cells (Invitrogen).

### Phylogenetic analysis

Phylogenetic analysis was performed utilizing 16S rRNA sequences of length similar to that obtained for TS-821. Sequences were chosen for comparison based on high sequence homology from the BLAST search results or as representatives of different genera within the phylum Cyanophyta. All sequences were obtained through GenBank. Once all taxa were chosen, the sequences were aligned via the ClustalX algorithm within MEGA 4.0 (Tamura et al., 2007). The sequence alignment was exported to Modeltest 3.7, which chose the most appropriate statistical model for the generation of a phylogenetic tree (Posada and Crandall, 1998). Following the suggested model chosen by Modeltest 3.7, MEGA 4.0 generated a phylogenetic tree with the following parameters: neighbor-joining method coupled with a maximum composite likelihood of nucleotides, complete deletions of all gaps and missing data within the sequence alignment, the heterogeneous pattern among lineages with a gamma parameter of 0.5087, and with 5000 bootstrapping replicates (Gordiichuk et al., 2014). The resulting tree was condensed to a consensus tree containing bootstrap values located on the branches.

### Cryo-EM data acquisition

Aliquots of 3 μl of PSI samples were applied on glow-discharged holey carbon grids (Quantifoil R2/2, 400 mesh) coated with a continuous 2-nm carbon film. The grids were blotted and plunge-frozen using a Vitrobot Mark III (Thermo Fischer Scientific). Cryo-EM micrographs were recorded at liquid nitrogen temperature on a Titan/Krios transmission electron microscope (Thermo Fisher Scientific, USA) operating at 300 kV at NeCEN (Leiden, the Netherlands). Micrographs were recorded at a nominal magnification of 130 000× using a K2 Summit direct electron detector (Gatan) using a pixel size of 1.108 Å, with a dose rate of ∼4.3 electrons/Å^2^/s and defocus values ranging from −0.6 to −3 μm. The total exposure time was 12.0 s, and intermediate frames were recorded at 0.5-s intervals resulting in an accumulated dose of ∼50 electrons per Å^2^ and a total of 24 frames per micrograph.

### Cryo-EM image processing and 3D reconstructions

The cryo-EM image processing was performed in SCIPION 2.0 (de la Rosa-Trevin et al., 2016) using the integrated protocols. A total of 4845 raw movies were corrected for beam-induced motion using MotionCorr2 (Zheng et al., 2017). A sum of all frames, filtered according to exposure dose, in each image stack was used for further processing. CTF parameters for each micrograph were determined by CTFFIND4 (Rohou and Grigorieff, 2015) and xmipp3 - ctf estimation (Sorzano et al., 2013, 2018). Particle selection was done using xmipp3 - manual-picking/xmipp3 - auto-picking (Sorzano et al., 2013, 2018). A total of 325 648 picked particles were extracted and underwent the 2D classification using xmipp3 - cl2D (Sorzano et al., 2010, 2018) classification protocols. After the xmipp3 - cl2D automated particle picking, each micrograph was revised by adding/removing missed and wrongly picked particles correspondently. The initial C2 symmetry enforced model was calculated *de novo* based on the good 2D class average projections obtained during the previous step, using xmipp3 - ransac (Vargas et al., 2014; Sorzano et al., 2018). The 3D classification was performed using the RELION 3.0 3D classification protocol (Scheres, 2016). The best 3D class outcome 122 411 particles were subjected to further refinement protocol using Xmipp highres (de la Rosa-Trevin et al., 2016; Sorzano et al., 2018, 2021). The resulting refined cryo-EM 3D map contained 66 130 particles. After 56 281 particles were removed during the processing as suboptimal, the 66 130 particles from the previous step were submitted for RELION 3.0 auto-refinement (Supplemental Figure 1). One cycle of Bayesian polishing and CTF refinement was done, followed by 3D refinement after each Bayesian polishing and CTF refinement step. The resultant cryo-EM map was post-processed, resulting in 3.72 Å average resolution. The local resolution was calculated using ResMap (Kucukelbir et al., 2014) showing the range of resolution from 3.2 Å to 6 Å (Supplemental Figure 2) and were sharpened using LocalDeblur (Ramirez-Aportela et al., 2020), using as input the resolution map calculated with Monores (de la Rosa-Trevin et al., 2016; Vilas et al., 2018). This cryo-EM map was used for model building and then underwent another round of sharpening using the sachselab - locscale (de la Rosa-Trevin et al., 2016; Jakobi et al., 2017) protocol. Reported resolutions are based on the gold-standard FSC using the 0.143 cutoff criterion (Supplemental Figure 2A). The local resolution was determined using DeepRes (Ramirez-Aportela et al., 2019) (Supplemental Figure 2B).

### Model building

The obtained cryo-EM map was used for manual model building in Coot using the single protomer of cyanobacterial PSI from Te-BP1 as a reference. The resolution of the map was sufficient to assign all protein subunits unambiguously and to model most of the Chl *a* molecules. Rounds of real-space refinement were performed in Phenix and included simulated annealing protocol and NCS restraints. Coordinates were manually edited in Coot after each refinement cycle and subjected to further rounds of refinement. The final validation check was performed with MolProbity and Phenix validation tools. Images were prepared with the open-source version of PyMol (https://sourceforge.net/projects/pymol/) and Chimera (https://www.cgl.ucsf.edu/chimera/).

### HADDOCK analysis

The cryo-EM model was refined with HADDOCK (Dominguez et al., 2003), and calculations were performed as previously described (Kastritis and Bonvin, 2010). The OPLS force field (Jorgensen et al., 1996) was used for topology and parameter file generation, and PRODRG (van Aalten et al., 1996) was used to parameterize the Chl *a* molecules. Energy calculations were performed for the two interfaces of the tetramer using the HADDOCK score along with its component energy terms. These terms include van der Waals (*E*_vdW_) and Coulomb (*E*_elec_) intermolecular energies representing non-bonded and electrostatic energies, an empirical desolvation term (*E*_desolv_) (Fernandez-Recio et al., 2004), and buried surface area upon complex formation in Å^2^.

### Monte Carlo simulations

Side-chain rotamers and protonation states were generated using the Multi-Conformer Continuum Electrostatics program (Song et al., 2009). Monte Carlo sampling was then used to generate Boltzmann distribution of the different conformations based on the electrostatic and van der Waals energies. The electrostatic interactions were calculated by solving the Poisson-Boltzmann equation using DELPHI software (Baker et al., 2001), and calculation of the van der Waals interactions was based on the Amber force field (Cornell et al., 1995).

### Central cavity analysis

The volume of the central cavity was calculated using the CASTp webserver (Tian et al., 2018a) with a probe radius of 1.4 Å. Only subunits that border the central cavity (PsaI, PsaL, and PsaM) were included due to file size limits on the webserver. CHARMM-GUI (Jo et al., 2008) was used to generate an MGDG, DGDG, SQDG, and DPPG mixed lipid bilayer with a lipid composition of 47:23:21:9 based on averages from previously reported cyanobacteria lipid compositions (Van Walraven et al., 1984; Murata, 1998; Kiseleva et al., 1999; Sakurai et al., 2006). The tetramer was then embedded in the membrane by removing lipids within 0.6 Å from the protein using VMD (Humphrey et al., 1996) to provide a rough estimate of how many lipids could fit into the central cavity.

### Data availability

The data supporting the findings of this article are available from the corresponding author upon reasonable request. A reporting summary for this article is available as a supplementary information file. Model coordinates and density maps are available in the Protein Data Bank (PDB: 6QWJ) and the EM Data Bank (EMD: 4659).

### Bioinformatics methods

#### Cyanobacterial genome selection

All 1639 cyanobacteria genomes available as of January 25, 2019, were downloaded from NCBI based on keyword searches with excluding viruses and phages. Based on the whole-genome sequences, a subset of these 1639 genomes was generated by building a Mash tree (Ondov et al., 2016) to reduce redundancy. We selected a cutoff value of 0.1 based on the first plateau in the plot (Supplemental Figure 1), which resulted in 295 clusters. In some of these clusters, we observed sets of highly related or even identical organisms (data not shown), so we selected a unique yet representative member from each of the 295 clusters using random number generation from the Python package NumPy (Jones et al., 2001). This allowed us to randomly choose a single genome from each cluster yet also significantly reduced redundancy from 1639 to only 295 distinct non-redundant organisms.

#### PsaL protein identification and selection

The genes within these 295 genomes were then annotated using Prokka (Seemann, 2014) with -Cdsrnaolap and default options. From this annotated set of 295 genomes, BLAST 2.7 (Altschul et al., 1990) was used to identify PsaL orthologs using the PsaL sequence from *T.e*. BP-1 (UniProt: Q8DGB4). Initially, over 1567 putative PsaL orthologs were identified. The list of putative orthologs was reduced by filtering based on sequence identity (≥40%), sequence coverage (≥35%), and e-value (≤1e^−10^), resulting in 113 orthologs. These heuristic values were determined by plotting the percent identity range on the *x* axis from 0 to 100 and the number of hits meeting the criteria on the *y* axis for all PsaL versus 295 genome sequences while keeping e-value of ≤1e^−10^ and sequence length difference of ≥35. These filters reduced the analysis to PsaL orthologs with a percent identity cutoff of ∼40%.

#### Phylogenetics and motif analysis

Using these 113 PsaL orthologs, the sequences were aligned using MUSCLE v3.8.31 (Edgar, 2004) using default options. The genomes were further reduced to 83 representatives in total by visually inspecting the alignments in Jalview (Clamp et al., 2004), using the known anchors and selected genomes. The anchor genome for the far-red group was the far-red JSC-1 genome with a crystal structure available (Gan et al., 2014). The second filter applied was that all *psaL* sequences that were less than 180 amino acids long were included in analyses excluding the known *psaL* ortholog for JSC-1. The representative psaL orthologs were further reduced by the manual alignment of the sequences for each group, which removed outliers with length greater than the mean of the group analyzed. In addition, to these 83 randomly selected PsaL sequences we also included 17 experimentally verified tetramer-forming PsaL sequences (Li et al., 2019), 12 experimentally determined far-red forms of PSI (Gan and Bryant, 2015), and three well-characterized *Prochlorococcus* strains—SS120, MIT9313, and MED4 (MacGregor-Chatwin et al., 2019). The trimeric group was selected based on manual alignment and visual inspection of genomes that skewed alignment from known *psaL* sequence in the group from the trimer anchor genome *T.e*. BP-1 and *Syn* PCC 6803. The tetramer group was selected based on manual alignment and visual inspection of genomes that skewed alignment relative to the tetramer anchor genome's *psaL* sequence, *Chroococcidiopsis* sp. TS-821. The marine *Prochlorococcus/Synechococcus* group had three anchors, *Prochlorococcus* strain SS120, MIT9313, and MED4 (MacGregor-Chatwin et al., 2019). With these selected 128 proteins, a maximum-likelihood tree was built using FastTree Version 2.1.11 with default options. The four semi-monophyletic groups formed in the tree include members from four distinct groups: 18 trimeric PSI; 28 far-red PSI; 36 tetrameric PSI; and 26 marine PSI. For each group, a linker region and sequence LogoPlots for each group were generated by WebLogo (Crooks et al., 2004).

## Funding

Support has been provided by the 10.13039/100015605Gibson Family Foundation, the Bredesen Center for Interdisciplinary Research and Education, the Dr Donald L. Akers Faculty Enrichment Fellowship, and 10.13039/100000001National Science Foundation (DGE-0801470 and EPS-1004083) support to B.D.B. In addition, J.M. and B.D.B. have been supported via a JDRD award from University of Tennessee (10.13039/100014455UTK)/Oak Ridge National Lab Science Alliance to B.D.B., M.L. has been supported as a CIRE Fellow at 10.13039/100007135University of Tennessee, Knoxville, C.J.C. was supported by a UTK NSF GFRP award (grant no. 2017219379), and K.S. has been supported by the Tickle College of Engineering, UTK.

## Author contributions

B.D.B. and E.J.B. conceived the project; D.A.S. and B.D.B. led the experimental design; M.L. performed cell culturing and PSI isolations; D.A.S. performed TEM analysis; D.A.S. performed 2D particle analysis and 3D reconstruction; D.A.S., C.O.S.S., E.R.-A., and P.L.K. performed density mapping and refinement; A.G. performed model building and refinement; D.A.S. and P.L.K. performed HADDOCK calculations; M.A. performed Monte Carlo simulations. Structure analysis and comparison was guided by B.D.B. and performed by J.M. and C.J.C. Genomic and bioinformatics analysis was performed by K.S. Figures were made by D.A.S., J.M., C.J.C., and B.D.B. The manuscript was written by D.A.S., J.M., C.J.C., and B.D.B. with input from all other authors.

## Supplemental information

Document S1. Supplemental Figures 1–9 and Supplemental Tables 1 and 2

Document S2. Article plus supplemental information
