# Supplementary material for: Cryo-EM structure of a tetrameric photosystem I from Chroococcidiopsis TS-821, a thermophilic, unicellular, non-heterocyst-forming cyanobacterium
Source: Plant Commun. 2021 Oct 13;3(1):100248. doi: 10.1016/j.xplc.2021.100248 (PMC8760143; doi:10.1016/j.xplc.2021.100248)
Supplement: Document S1. Supplemental Figures 1–9 and Supplemental Tables 1 and 2 [file mmc1.pdf]

Plant Communications, Volume *3*

Supplemental information

Cryo-EM structure of a tetrameric photosystem I from *Chroococcidiopsis* TS-821, a thermophilic, unicellular, non-heterocyst-forming cyanobacterium

Dmitry A. Semchonok, Jyotirmoy Mondal, Connor J. Cooper, Katrina Schlum, Meng Li, Muhamed Amin, Carlos O.S. Sorzano, Erney Ramírez-Aportela, Panagiotis L. Kastritis, Egbert J. Boekema, Albert Guskov, and Barry D. Bruce

Supplemental Information

Cryo-EM Structure of a Tetrameric Photosystem I from *Chroococcidiopsis* TS-821, a Thermophilic, Unicellular, Non-heterocyst-forming Cyanobacteria.

Supplemental Figures

Fig. S1. Cryo-EM data collection, 2D classification and 3D model reconstruction

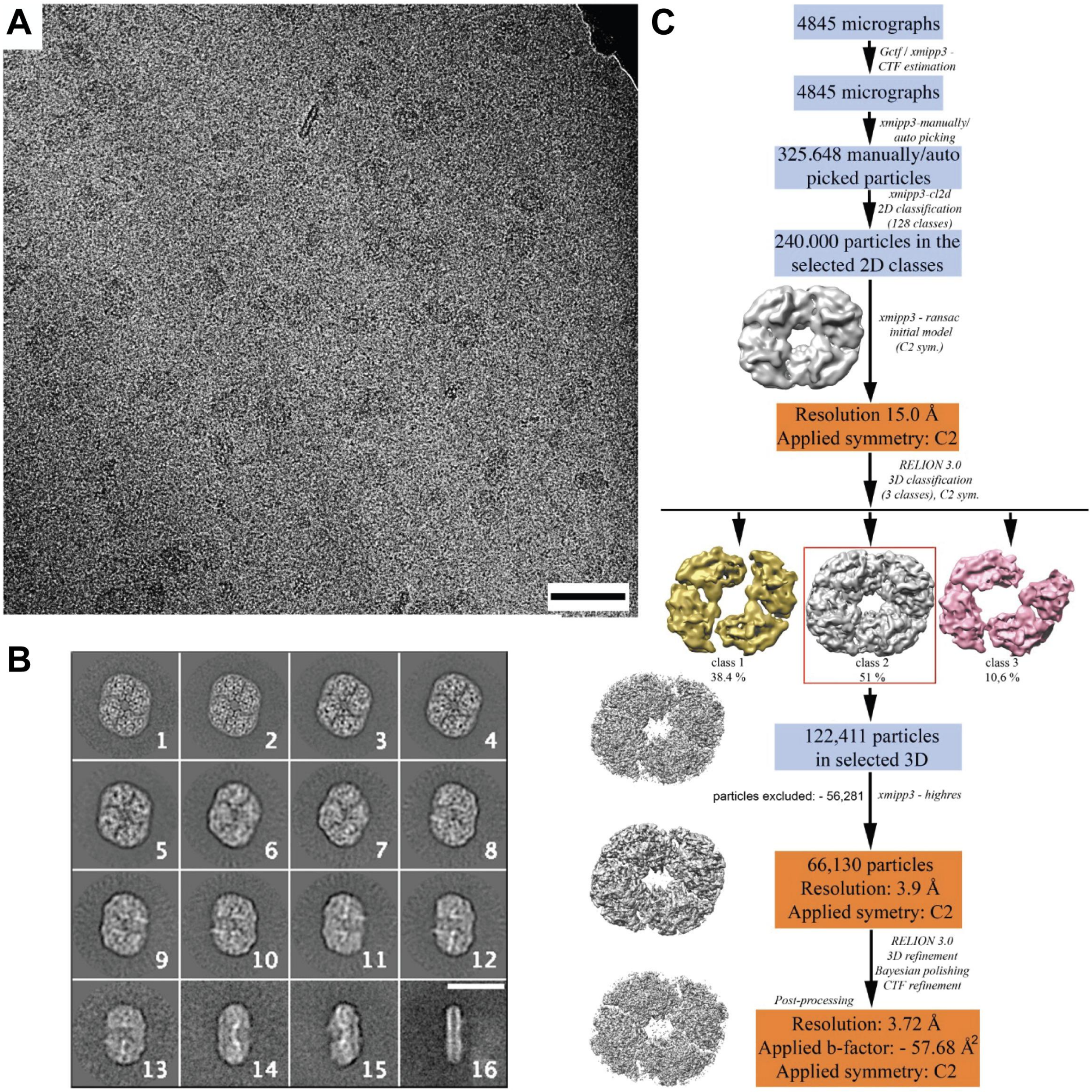

A) Typical motion corrected cryo-TEM micrographs of the TS-821 PSI tetramer (scale bar = 50 nm). **B)** Representative 2D class averages of PSI tetramer particles processed by xmipp3-cl2D protocol (scale bar = 25 nm). **C)** A schematic flowchart showing the

classification scheme for the PSI tetramer complex. The PSI tetramer structure was reconstructed at 3.72 Å resolution from 66.130 particles. See Method section for more details.

Fig. S2. Evaluation and verification of the cryo-EM 3D map quality

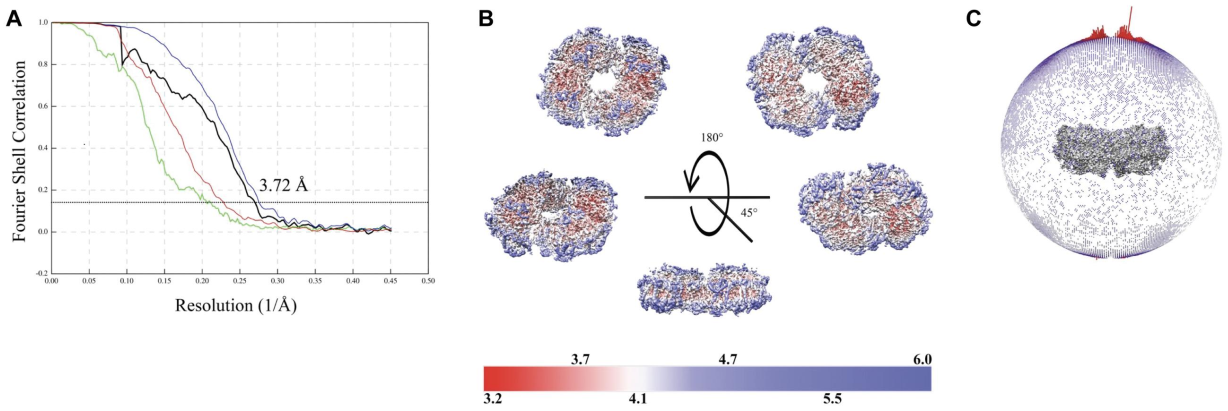

A) Fourier shell correlation curves of the PSI tetramer calculated after the post-processing step, where the green curve is FSC unmasked maps, red – FSC phase randomized masked maps, black - FSC corrected and blue – FSC masked maps. **B)** Local resolution maps of the PSI tetramer. **C)** Angular distribution of the particles used for reconstruction of PSI tetramer complex. Each cylinder represents one view, and the height of the cylinder is proportional to the number of particles for that view.

Fig. S3. Structure and stability of the two-dimer interface

A

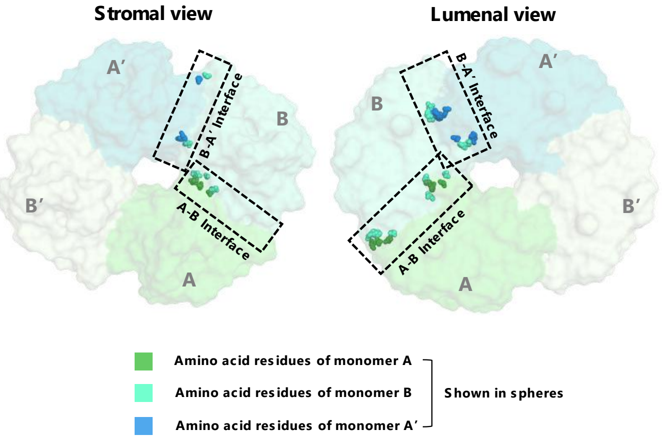

B

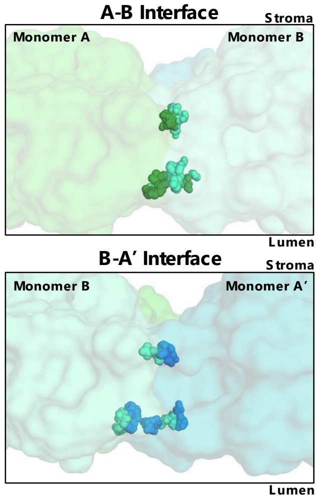

C

A-B Interface

Monomer A			Monomer B			ΔEnergy
Chain	Amino Acid	Res #	Chain	Amino Acid	Res #	(kcal/mol)
K	Ile	91	B	Leu	222	-1.24
L	Trp	45	I	Ile	39	-1.05
L	C-term	172	B	Pro	94	-0.86
L	Phe	171	I	Phe	6	-0.83
K	Asn	88	B	Thr	214	-0.64
L	Ile	57	L	Asn	140	-0.63
L	Arg	61	L	Ser	144	-0.57
L	Arg	54	L	Ala	114	-0.46
L	Trp	45	I	C-term	39	-0.42
L	Leu	166	L	Leu	96	-0.37
L	Arg	61	L	Ser	144	-0.36
K	Lys	93	B	Arg	223	-0.28
K	Ile	91	B	Arg	223	-0.28
K	Ile	91	B	Phe	225	-0.27
Total						-8.27

B-A′ Interface

Monomer B			Monomer A′			ΔEnergy
Chain	Amino Acid	Res #	Chain	Amino Acid	Res #	(kcal/mol)
L	Trp	45	B	Phe	161	-2.26
A	Leu	493	B	Arg	223	-1.56
A	Ile	490	B	Phe	225	-0.73
A	Thr	498	B	Phe	226	-0.69
L	Phe	171	B	Asp	210	-0.60
A	Ser	497	B	Phe	226	-0.55
L	Trp	45	B	Leu	155	-0.54
L	Lys	172	B	Thr	131	-0.47
L	C-term	172	B	Ser	137	-0.28
L	C-term	172	B	Asn	133	-0.28
L	C-term	172	B	Asn	133	-0.27
A	Ile	490	B	Arg	233	-0.26
K	Ile	43	B	Phe	311	-0.26
Total						-8.75

D

		A-B Interface	B-A′ Interface
# Amino Acids		78	119
# Proximal Chl (<24 Å)		A(6); B(5)	B(3); A'(3)
# Proximal Carotenoids		4	3
# Flanking TMD (<12 Å)		7	14
Flanking TMDs		A-PsaB-2; A-PsaB-5; A-PsaI; A-PsaM; A-PsaL-1; B-PsaL-1	
Buried Surface Area ($Å^2$)	- Chl	1581	914
	+ Chl	2418	1564
Electrostatic interactions (kcal/mol)	- Chl	-17.6	-28.5
	+ Chl	-13.8	-38.3
Electrostatic surface interactions (kcal/mol)		-0.4	0.1
Van der Waals surface Interactions (kcal/mol)		-14.0	-16.0
Total surface interactions (kcal/mol)		-14.4	-15.9

The stability of interfaces in tetrameric PSI. (A) Two distinct interfaces are observed in tetramer (labeled as A-B and A’-B); each individual PSI is colored differently. The residues that contribute significantly to the stabilization energy are shown as spheres. This contribution is dominated by the dispersion forces (B) The close up of the two interfaces, A-B and B-A’. (C) the dispersion energies contribution of individual amino acids into the stabilization of interfaces calculated using Amber forcefield. (D) The electrostatics and van der Waals energies and the accessible surface areas of both interfaces.

Figure S4: Comparing BCRs in individual monomers of TS-821 (PDB ID- 6QWJ) and *Nostoc* (PDB ID-6JEO):

A

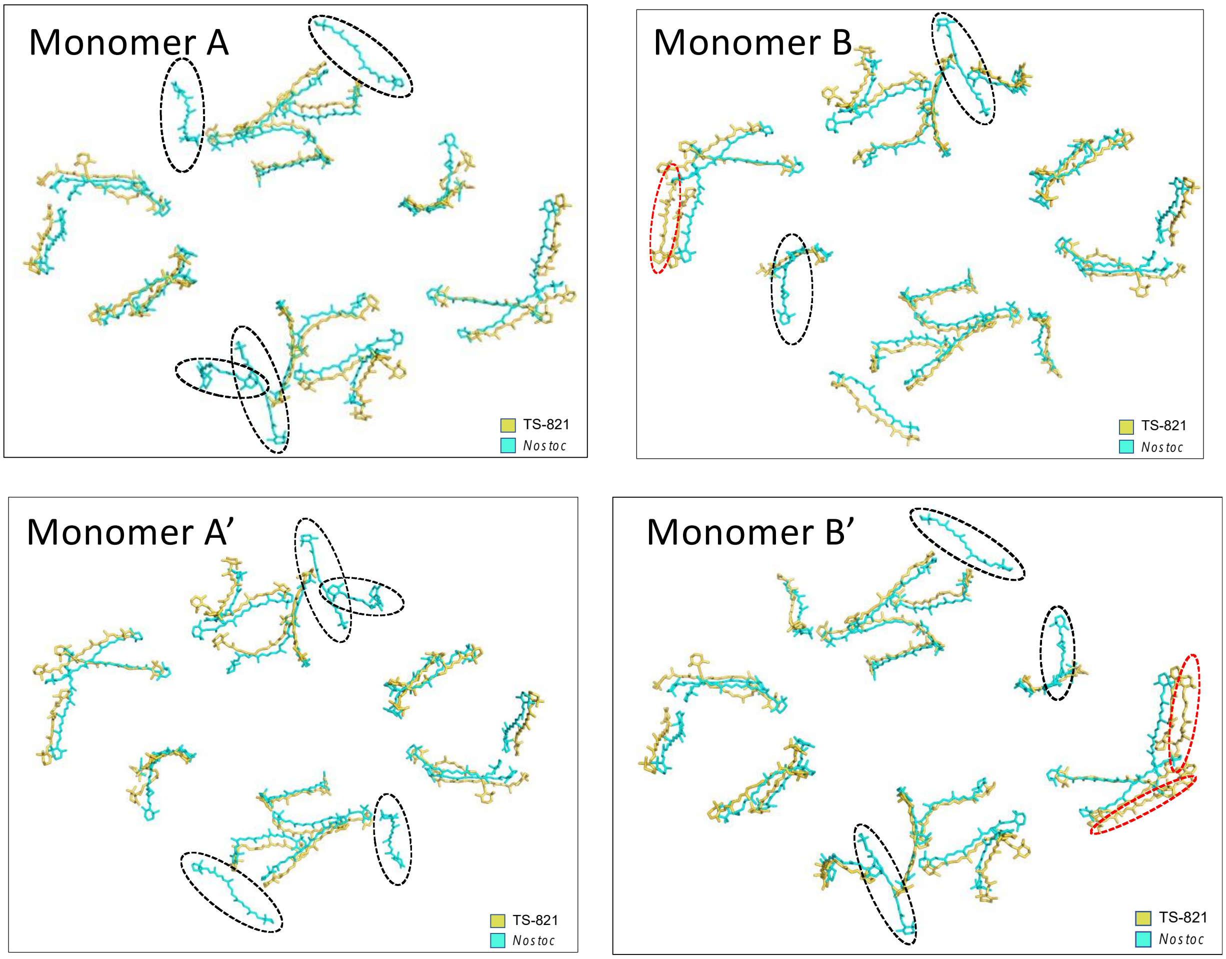

B

Species	Monomer A	Monomer B	Monomer A'	Monomer B'
TS-821	18	21	18	21
Nostoc	22	22	22	22
T. e. BP-1	22#	22#	22#	N/A

#The monomers of the *T. e.* PSI trimer (1JB0) are identical

(A) The BCRs are shown as sticks (TS-821- yellow, Nostoc- cyan). The monomers are aligned individually, for example, monomer A of TS-821 is aligned with monomer A of

Nostoc. The RMSD values for alignment of individual pairs of monomers A, B, A' and B' from both species are 2.356, 2.396, 2.325 and 2.393 Å, respectively. The dashed black ovals indicate additional BCRs in *Nostoc* while the dashed red ovals are additional BCRs in TS-821. **(B)** Table summarizing the BCR counts in TS-821 (PDB ID- 6QWJ), *Nostoc* (PDB ID-6JEO) and *T.e.* BP-1 (PDB ID- 1JB0).

Figure S5: Comparing Chl *a* in individual monomers of TS-821 (PDB ID- 6QWJ)

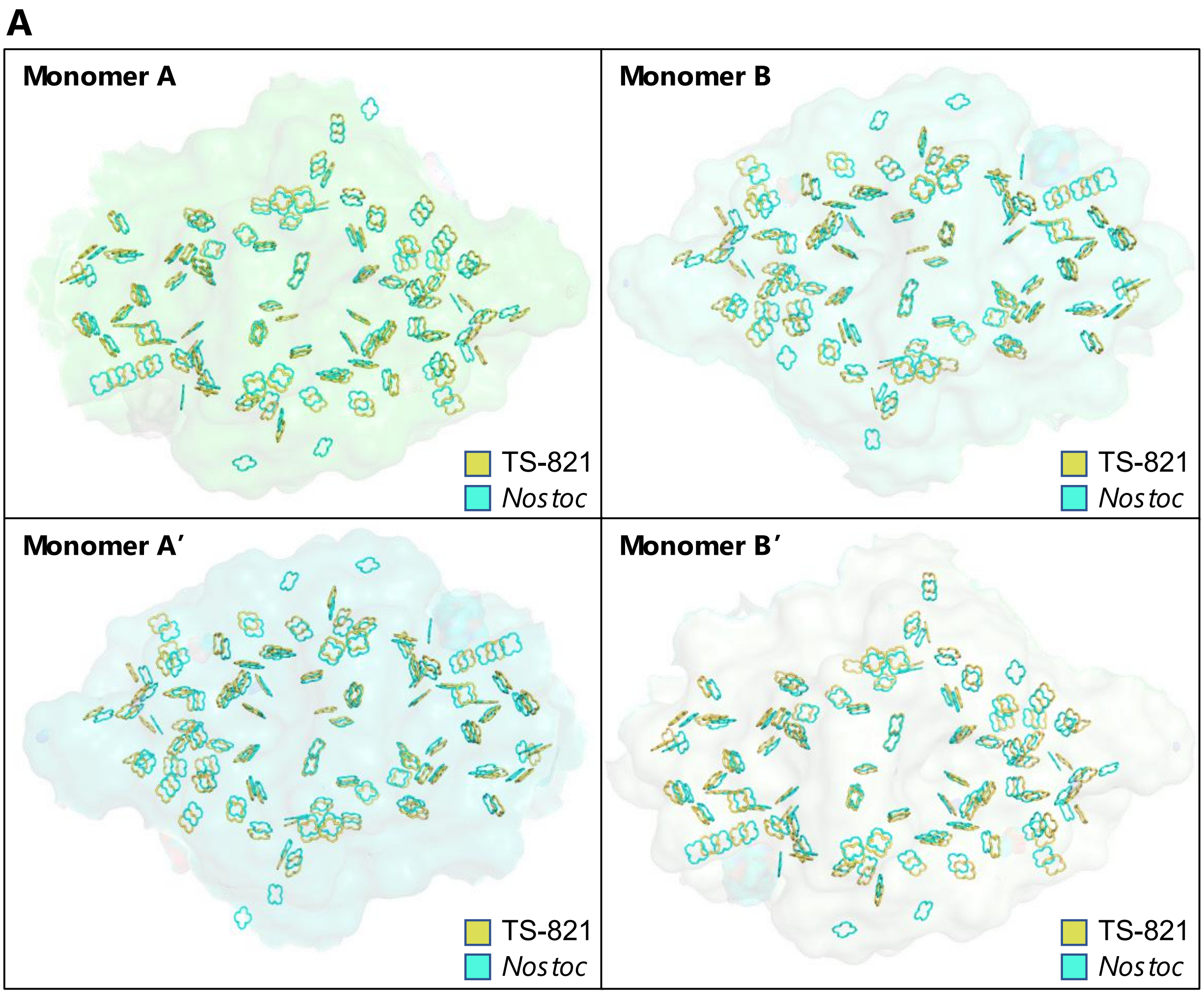

B

Species	Monomer A	Monomer B	Monomer A′	Monomer B′	Total
TS-821	91	89	90	88	358
Nostoc	96	94	96	94	380

and *Nostoc* (PDB ID-6JEO):

(A) The Chl *a* pyrrole rings are shown in sticks (TS-821- yellow and *Nostoc*- cyan). The surface represents each monomer which are aligned individually, for example, monomer A of TS-821 is aligned with monomer A of *Nostoc*. The RMSD values for alignment of individual pairs of monomers A, B, A' and B' from both species are 2.356,

2.396, 2.325 and 2.393 Å, respectively. **(B)** Table summarizing the Chl *a* count in TS-821 (PDB ID- 6QWJ), *Nostoc* (PDB ID-6JEO).

Fig. S6. Distances between Mg^{2+}-Mg^{2+} of adjacent pyrrole rings of Chl *a*.

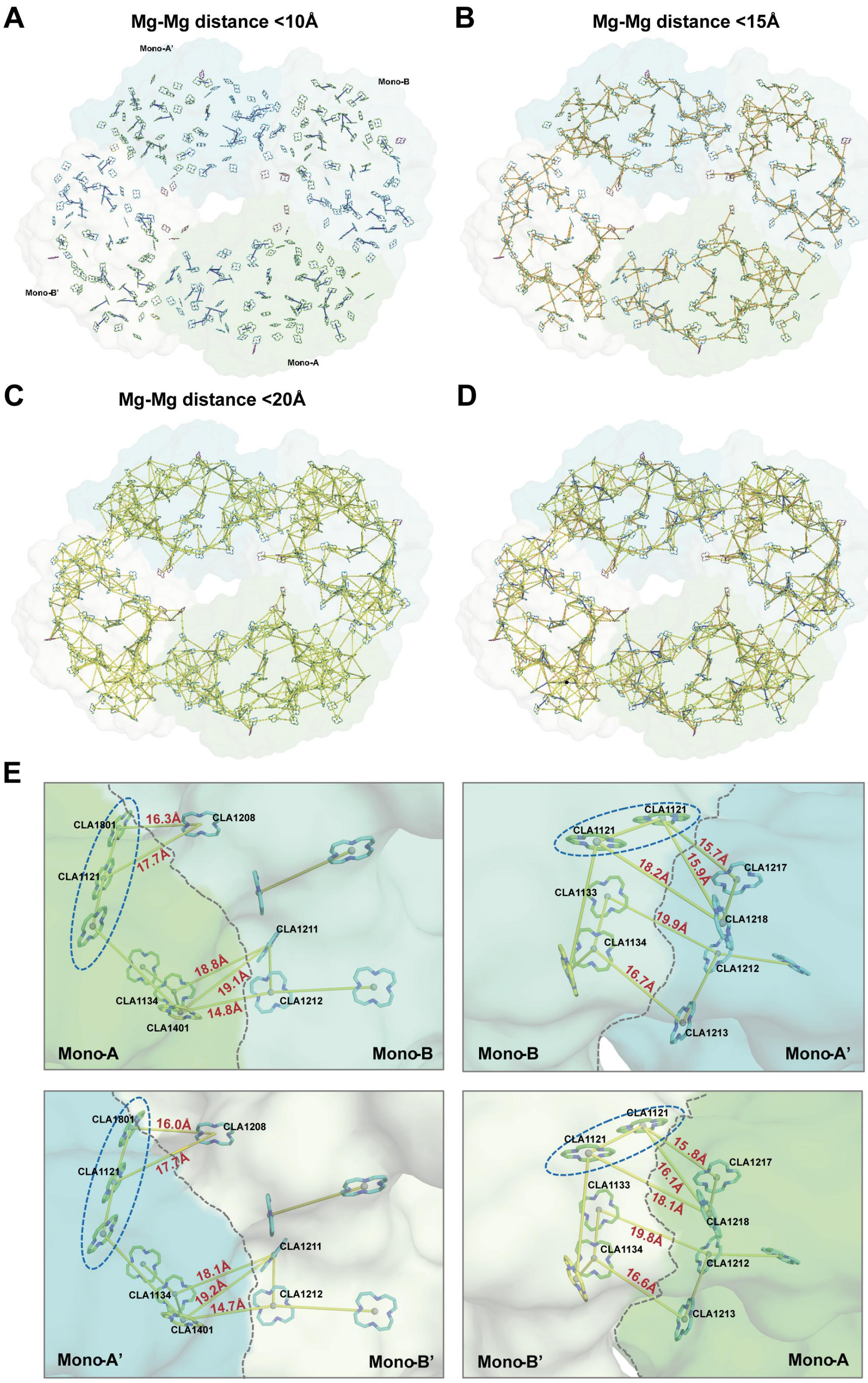

<u>Distances between Mg^{2+}-Mg^{2+} of adjacent pyrrole rings of Chl *a*</u>: Mg-Mg distances within (**A**) 10 Å (blue), (**B**) 15 Å (orange), and (**C**) 20 Å (yellow) (**D**) Combined Mg-Mg

distances (10, 15, and 20 Å). (**E**) Mg-Mg distances of Chl *a* molecules at the interface between PSI monomers: Chl *a* pyrrole rings are colored based on the associated subunit - green for PsaA, blue for PsaB and yellow for PsaK. The distances (between 12-20Å) as between Chls participating in interface are shown in red (Also see Table S2). The surface depicts the respective monomers (labelled in the bottom) separated by dashed gray line.

Supplemental Figure S7: Comparing lipids in TS-821 and *Nostoc*:

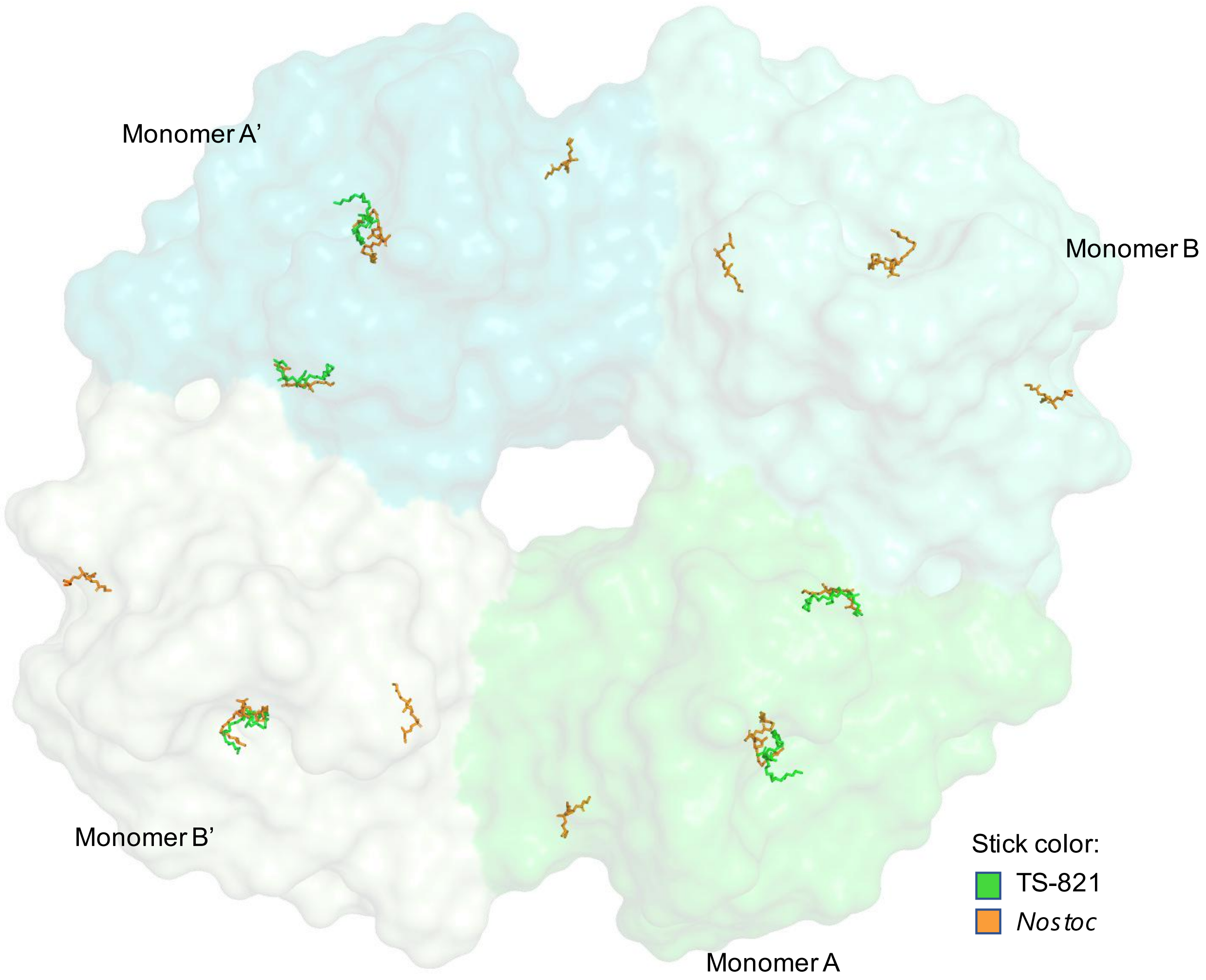

The lipids resolved, 1,2-dipalmitoyl-phosphatidyl-glycerole (LHG) are shown in green in TS-821 (PDB ID- 6QWJ) and aligned to *Nostoc* (PDB ID-6JEO) shown in orange.

Fig. S8. Comparison of Chl *a* arrangement of trimeric PSI (PDB-ID: 1JB0) from *T. e.* BP-1 and tetrameric PSI from TS-821

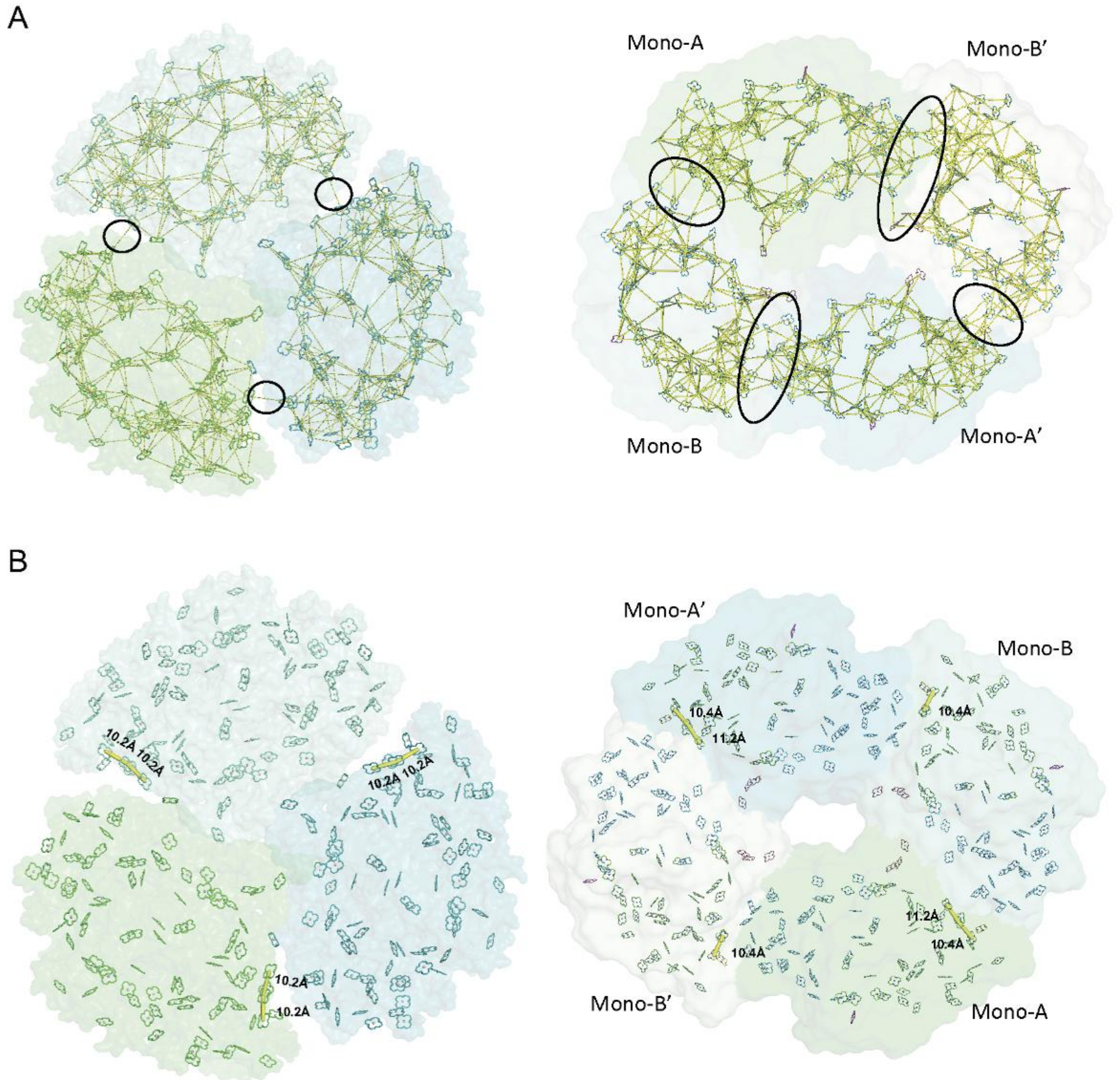

Comparison of Chl *a* arrangement of trimeric PSI (PDB-ID: 1JB0) from *T. e.* BP-1 and tetrameric PSI from TS-821: (**A**) The Chl a pyrrole rings are depicted in green and the distances (<20Å) between Mg-Mg are shown in yellow. Chl *a*'s at the interface between monomers are circled in black. **(B**) Distances between parallel Chl *a*'s at the interface of the monomers are shown and labelled.

Fig. S9. Alignment of tetrameric PSIs of TS-821 (PDB ID- 6QWJ) and *Nostoc* (PDB ID-6JEO)

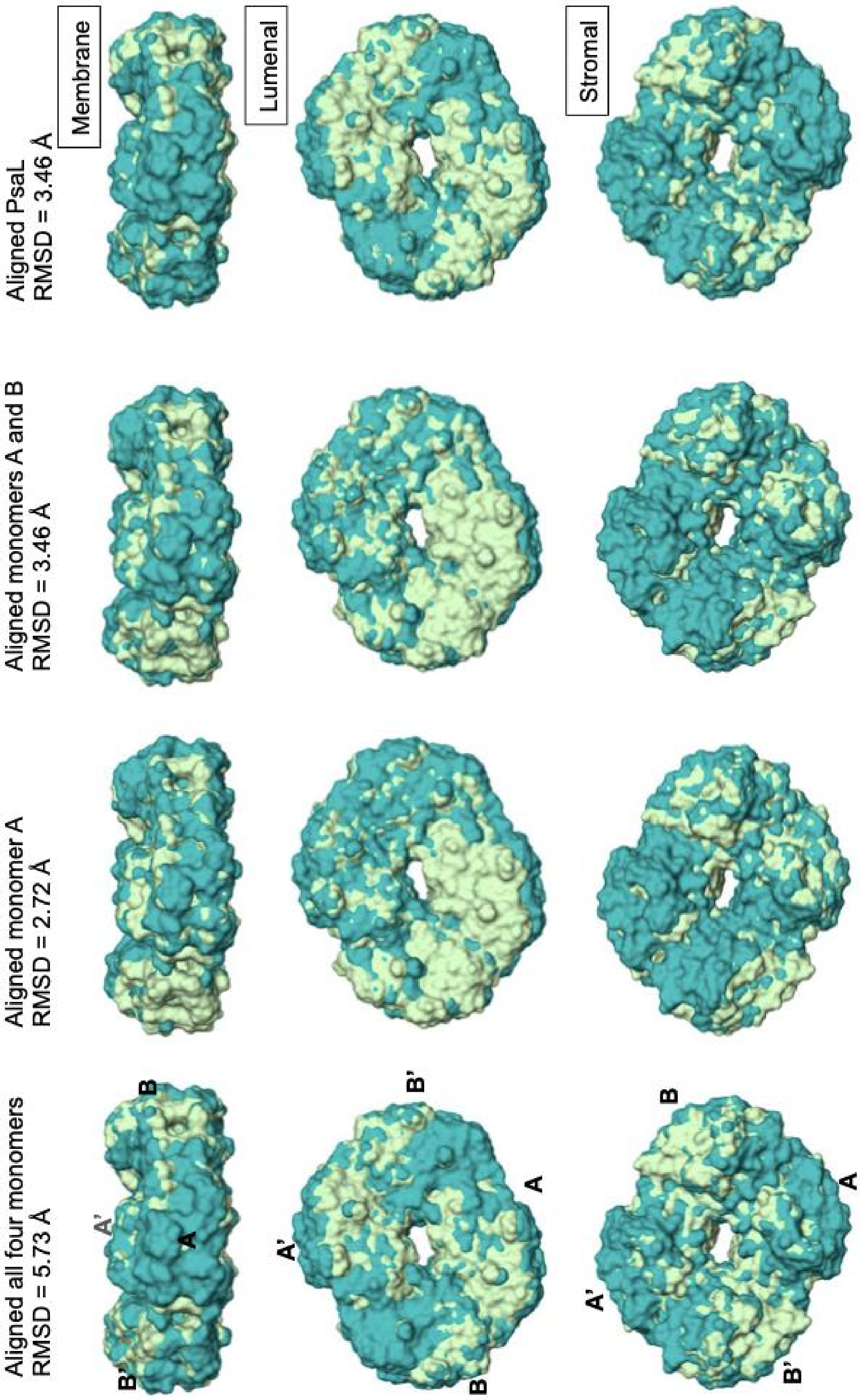

Alignment of tetrameric PSIs of TS-821(PDB ID- 6QWJ) and *Nostoc* (PDB ID-6JEO).

Comparing the tetramers of TS-821 and *Nostoc* by performing different alignment strategies- all four monomers; monomer A only; monomer A and B only; and PsaL. Pymol was used to generate these alignments.

Table S1. Statistics of data collection, processing and refinement.

**PDB ID**	6QWJ
**EMDB ID**	4659
**Data collection and Image processing**	
Microscope	FEI Titan Krios G2
Detector	K2 Summit direct electron detector (Gatan, Inc.)
Magnification	~130,000
Voltage (kV)	300
Defocus range (μm)	-0.6 to -3.0
Pixel size (Å/pix)	1.108
Total electron dose (e-/Å^2)	~50
Exposure time (s)	12
Number of frames per movie	24
Number of micrographs/movies	4,845
Initial particle images (no.)	325,648
Final particle images (no.)	66,130
Map resolution (Å)	3.9
Applied symmetry	C2
**Refinement**	
Initial Model used (PDB code)	1JBO
Gold-standard FSC threshold	0.143
Model resolution (Å)	3.9
No. of Protein subunits (total/tetramer)	44
No. of chlorophyll	358
No. of carotenoid	64
No. of phaeophytin	8
No. of Fe-S centers (4Fe-4S)	12
No. of Prosthetic groups/tetramer	448
No. of atoms (Protein)	69,176
No. of atoms (cofactors)	20,740
No. of atoms (total)	89,916
B-factor- protein (Å^2)	94.46
B-factor- Cofactors (Å^2)	114.27
Number of missing residues (from coding sequence)	209/2413
% Total amino acid placement	8.66
r.m.s. deviations- Bond lengths (Å)	0.005
r.m.s. deviations- Bond angles (°)	0.845
**Structure Validation**	
MolProbity Score	3.06
ClashScore	14.89
Rotomer Correctness (%)	89.90
**Ramachandra Plot Analysis**	
Favored (%)	87.33
Allowed (%)	12.53
Disallowed (%)	0.14
**Model vs Data**	
CCmask	0.79
CCbox	0.84
CCpeaks	0.70
CCvolume	0.79
Mean CC for ligands	0.80

Table S2. Table showing Mg-Mg distances of Chl *a* molecules at the interface between PSI monomers as depicted in Fig. S5-E.

Monomer	Chain	Residue ID	Monomer	Chain	Residue ID	Mg-Mg Distance (Å)
A	A	1121	B	B	1208	**17.7**
A	A	1134	B	B	1211	**18.8**
A	A	1801	B	B	1208	**16.3**
A	K	1401	B	B	1211	**19.1**
A	K	1401	B	B	1212	**14.8**
A	B	1212	B'	A	1133	**19.8**
A	B	1213	B'	A	1134	**16.6**
A	B	1217	B'	A	1121	**15.8**
A	B	1218	B'	A	1120	**18.1**
A	B	1218	B'	A	1121	**16.1**
B	A	1120	A'	B	1218	**18.2**
B	A	1121	A'	B	1217	**15.7**
B	A	1121	A'	B	1218	**15.9**
B	A	1133	A'	B	1212	**19.9**
B	A	1134	A'	B	1213	**16.7**
A'	A	1121	B'	B	1208	**17.7**
A'	A	1134	B'	B	1211	**18.9**
A'	A	1801	B'	B	1208	**16.0**
A'	K	1401	B'	B	1211	**19.2**
A'	K	1401	B'	B	1212	**14.7**
